# Inhibition of cancer progression by a novel *trans*-stilbene derivative through disruption of microtubule dynamics, driving G2/M arrest, and p53-dependent apoptosis

**DOI:** 10.1038/s41419-018-0476-2

**Published:** 2018-04-18

**Authors:** Pravat Kumar Parida, Barun Mahata, Abhisek Santra, Sohini Chakraborty, Zhumur Ghosh, Sanghamitra Raha, Anup Kumar Misra, Kaushik Biswas, Kuladip Jana

**Affiliations:** 1 Division of Molecular Medicine, Bose Institute, P1/12, C.I.T. Scheme VIIM, Kolkata, West Bengal 700054 India; 2 The Bioinformatics Center, Bose Institute, Kolkata, West Bengal 700054 India; 3 Saha Institute of Nuclear Physics, Kolkata, India

## Abstract

Resveratrol, a *trans*-stilbene polyphenolic compound and its synthetic analogs are widely used bioactive molecules due to their remarkable chemo-preventive potential. Here, we have identified a novel synthetic *trans*-stilbene compound, Z-DAN-11 ((Z)-3-(3, 4-dimethoxyphenyl)-2-(3, 4, 5-trimethoxyphenyl) acrylonitrile) which shows remarkable efficacy in blocking tumor growth and progression both in vitro and in vivo. Z-DAN-11 inhibits proliferation of cancer cells in vitro through microtubule depolymerization that induced G2/M arrest and consequently leads to apoptotic cell death. More importantly, Z-DAN-11 shows limited cytotoxicity to normal cells as compared to cancer cells. Quite interestingly, we have found that Z-DAN-11-mediated ROS production helps in dramatic alteration in the mitochondrial redox status which critically contributes to the apoptosis. Mechanistic studies reveal that Z-DAN-11 induces the expression of pro-apoptotic proteins and decreases anti-apoptotic protein expression that decisively helps in the activation of caspase 8, caspase 9, and caspase 3, leading to cleavage of PARP1 and cell death via intrinsic and extrinsic pathways of apoptosis. Moreover, Z-DAN-11-mediated apoptosis of cancer cells is through a partial p53-dependent pathway, since both HCT116 p53^−/−^ cells as well as p53-silenced cells (siRNA) were able to block apoptosis partially but significantly. Importantly, Z-DAN-11 also imparts its anti-tumorigenic effect by inhibiting clonogenic property and anchorage-independent growth potential of cancer cells at concentrations at least 10 times lower than that required for inducing apoptosis. Finally, in vivo study with immuno-competent syngeneic mice shows Z-DAN-11 to be able to impede tumor progression without any adverse side-effects. Hence, we identified a novel, synthetic *trans*-stilbene derivative with anti-tumorigenic potential which might tremendously help in devising potential therapeutic strategy against cancer.

## Introduction

Among the various interventions used therapeutically against cancer, chemotherapy along with radiotherapy has been the most widely used and potential strategies for treating this disease^1,2^. Although, an ideal chemotherapeutic drug should target a cancer cell by acting against a cancer-specific receptor, protein or DNA, in reality, however, chemotherapeutic applications are limited primarily owing to their adverse effects on the normal cells^3,4^. This has increasingly led to studies aimed towards identification of novel compounds, preferably from natural sources having anti-cancer potential with minimal collateral damages to normal cells. Among them, polyphenols, many of which are found to be abundantly available in edible fruits, have shown promise^5,6^; however, only a few of these went past laboratory studies to being potentially therapeutic against cancer^7^. In recent years, *trans*-stilbene polyphenolic compounds like Resveratrol have gained tremendous attention due to their diversified biological effects^8–10^. Several methoxy and hydroxy derivatives of *trans*-stilbene have shown to possess anti-cancer activity in various human cancer cells through growth suppression by inducing cell cycle arrest and apoptosis^11–13^. Hence, the need to identify potential leads that can block cancer progression through targeted blocking of cells in particular phase of cell cycle^14^.

In the present study, we synthesized a number of *trans*-stilbene derivatives in an attempt to identify potential anti-cancer leads which is able to deliver a multi-modal yet targeted anti-cancer therapy, through affecting some of the key cellular processes primarily involved in carcinogenesis. Here, we evaluated the anti-cancer potential of 22 synthetic *trans-*stilbene derivatives using several cancer cell lines and identified one lead, Z-DAN-11 ((Z)-3-(3, 4-dimethoxyphenyl)-2-(3, 4, 5-trimethoxyphenyl) acrylonitrile), based on its ability to inhibit proliferation of cancer cells but not normal cells. Z-DAN-11 caused G2/M arrest in cancer cells, primarily by interfering with tubulin polymerization leading to disruption of microtubule formation. Furthermore, Z-DAN-11 was also able to induce genomic instability by DNA damage and consequent activation of p53 leading to activation of the caspase cascade eventually inducing apoptosis. In vivo, Z-DAN-11 demonstrates dose-dependent reduction in tumor burden and tumor progression in Balb/c mice without any significant hepatotoxicity. Hence, *trans-*stilbene derivative, Z-DAN-11 holds tremendous potential as an ideal lead towards targeted cancer chemotherapy, which either alone or in combination with existing chemotherapy may be used as a potential strategy towards cancer treatment.

## Results

### Synthesis and comparative cytotoxic efficacies of *trans-*stilbene derivatives

(Z)-2, 3-diarylacrylonitrile (*trans-*stilbene) derivatives were synthesized as described earlier^8^. Primarily, the series of compounds (Z-DAN-1 to Z-DAN-22) shown in Fig. S1 were assessed for their ability to block proliferation of HeLa, MCF-7 (cancer cells), and NKE cells (normal kidney epithelial cell). The anti-proliferative efficacies of the tested compounds in terms of IC_50_ were presented in Table S1, which showed three compounds Z-DAN-10, Z-DAN-11, and Z-DAN-12 to possess higher efficacy in inhibiting proliferation of HeLa as well as MCF-7 cells compared to the other derivatives. However, Z-DAN-11 displayed the highest selectivity index (SI) in both MCF-7 (SI = 7.26 ± 0.43) and in HeLa (8.08 ± 0.58) cells over Z-DAN-10 and Z-DAN-12. Thus, based on these results Z-DAN-11 was selected as a potent anti-cancer lead.

### Z-DAN-11 demonstrates selective cytotoxicity against several cancer cell lines

**Fig. 1:**
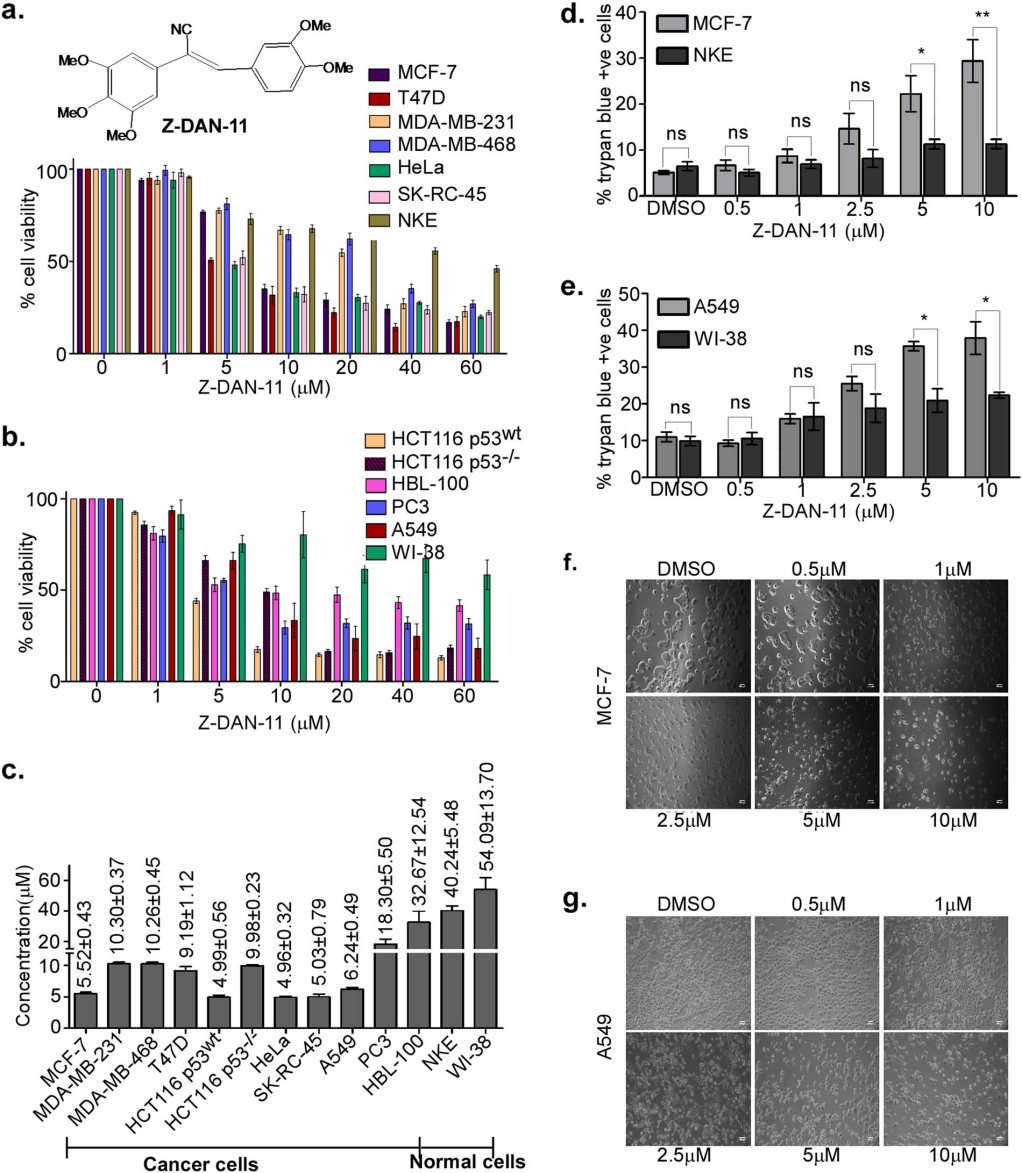
Z-DAN-11 demonstrates selective cytotoxicity against several cancer cell lines. In vitro cytotoxicity was measured by MTT assay in presence of Z-DAN-11 (0–60 µM) in various cell lines including **a** MCF-7, T47D, HeLa, SK-RC-45, MDA-MB-231, MDA-MB-468, NKE, and **b** HCT116 p53^wt^, HCT116 p53^−/−^, A549, HBL-100, PC3, and WI-38. **c** Bar graph showing comparative IC_50_ values of Z-DAN-11 in various cancer cells (MCF-7, T47D, HeLa, SK-RC-45, MDA-MB-231, MDA-MB-468, HCT116 p53^wt^, HCT116 p53^−/−^, A549, HBL-100, PC3) and normal cells (NKE, WI-38). **d**,** e** Dose-dependent increase in trypan blue positivity indicating significantly higher cytotoxic ability of the Z-DAN-11 in MCF-7 and A549 cells, as compared to normal cells NKE and WI-38 respectively. **f**,** g** Photomicrographs showing dose-dependent (0–10 µM) anti-proliferative effect of Z-DAN-11 in MCF-7 and A549 cells respectively. For all experiments, 0.2% DMSO served as vehicle control. All the data represent mean ± SEM of minimum three independent experiments (*p < 0.05**p < 0.01, ***p < 0.001, ns not significant)

Based on its high SI, Z-DAN-11 was further tested in an array of cell lines including T47D, HBL-100, MDA-MB-231, MDA-MB-468 (breast cancer cells), SK-RC-45 (renal carcinoma), HCT116 p53^wt^, HCT116 p53^−/−^ cells (colon cancer), PC3 (prostate cancer), A549 (lung carcinoma) as well as WI-38 (lung fibroblast) cells as shown in Fig. 1a–c. Data shows differential yet significant cytotoxicity in most cancer cell lines, relative to the normal cells, NKE or WI-38 (Fig. 1a–c). Interestingly, Z-DAN-11 showed limited cytotoxicity in HBL-100 (IC_50_ = 32.67 ± 12.54 µM) and PC3 cells (18.30 ± 5.50 µM). Further dose-dependent increase in trypan blue positivity indicated significantly higher cytotoxic ability of Z-DAN-11 in MCF-7 and A549 cells, as compared to normal cells NKE or WI-38 (Fig. 1d,e), respectively. Notably, the cytotoxicity of Z-DAN-11 at higher concentrations (5 µM and 10 µM) was significantly higher in cancer cells, when compared to normal cells (Fig. 1d,e), indicating the selective ability of Z-DAN-11 to induce cancer cell death. Additionally, the photomicrographs confirmed the concentration-dependent increase in sensitivity of MCF-7 and A549 cells towards Z-DAN-11 as shown in Fig. 1f,g. Altogether these results provide strong evidence for Z-DAN-11 in possessing selective cytototoxicity towards cancer cells.

### Z-DAN-11 interferes with cell cycle progression by inducing G2/M arrest

**Fig. 2:**
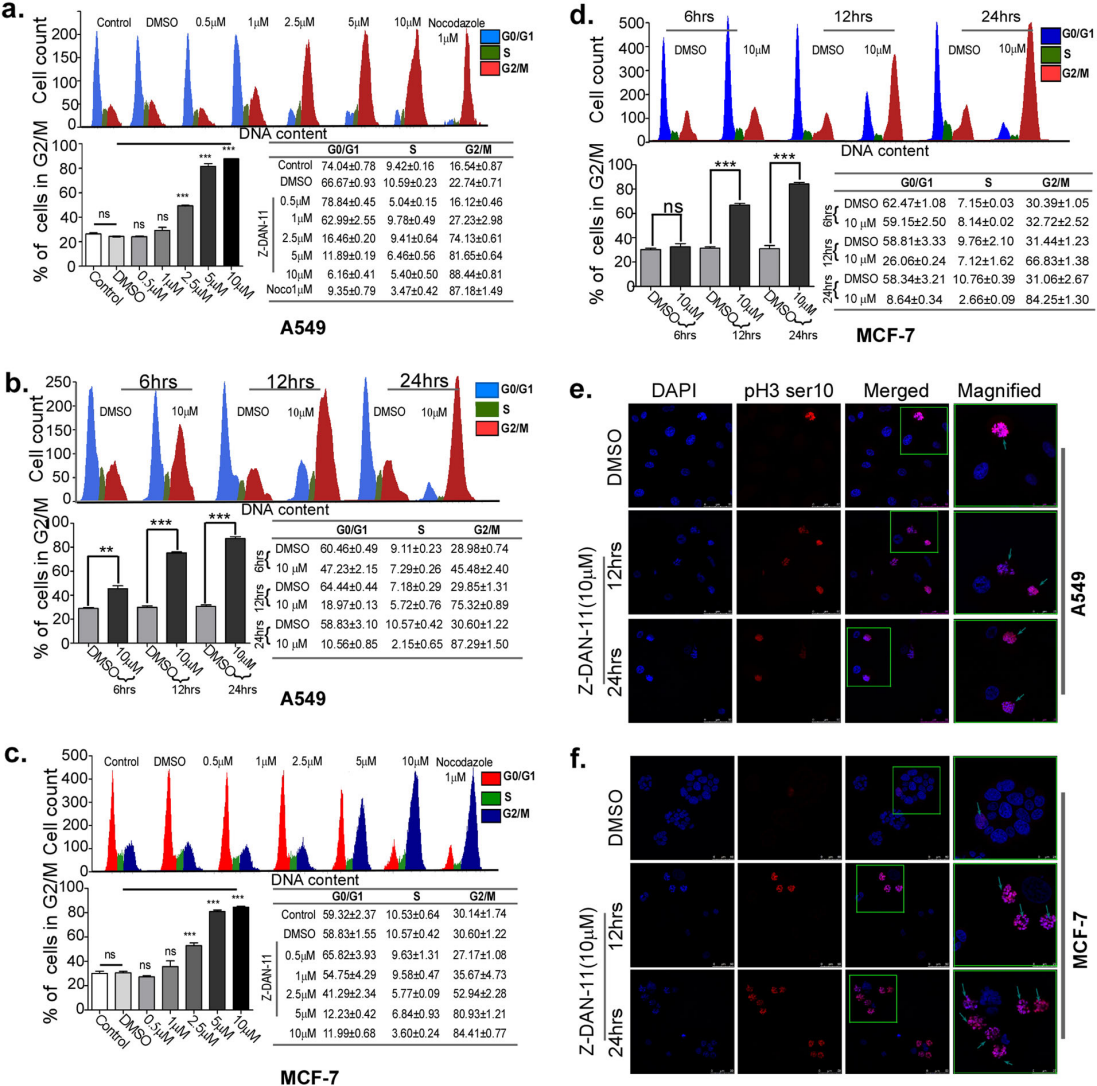
Z-DAN-11 interferes with cell cycle progression by inducing G2/M arrest. Double thymidine blocked synchronized A549 and MCF-7 cells were treated with Z-DAN-11 in a dose-dependent (0, 0.5, 1, 2.5, 5, and 10 µM) as well as time-dependent (6, 12, 24 h) manner. Post treatment, cells were harvested and fixed with ethanol followed by PI staining to check cellular distribution in different phases of the cell by flow cytometry. Histograms representing dose-dependent and time-dependent increase in the proportion of A549 cells **a**,** b** and MCF-7 cells **c**,** d** in G2/M phase along with decrease in G1 phase in response to Z-DAN-11. One-way ANOVA followed by Dunnett’s *t*-test was performed to comparisons of multiple group means (treatments) versus DMSO control. Data are representative of at least three independent experiments and bar graph shows mean ± SEM (*p < 0.05**p < 0.01, ***p < 0.001, ns not significant). Effect of Z-DAN-11 on mitotic DNA condensation marker p-H3 ser10 proteins in **e** A549 and **f** MCF-7 cells. Here, cells were incubated with Z-DAN-11 (10 μM) for the indicated time points and fixed with 3.7% formaldehyde. Post fixation cells were permeabilized with triton-X and incubated with anti-p-H3 ser10 primary antibody followed by incubation with Alexa-Fluor 594-conjugated secondary antibody. Counter staining was done using DAPI to visualize the nuclei. Images were acquired in Leica confocal microscope (Magnification: ×40)

Cycle distribution profile of various cancer cells in response to Z-DAN-11 clearly showed dose-dependent and time-dependent induction of G2/M arrest in both A549 (Fig. 2a,b) and MCF-7 cells (Fig. 2c,d). Interestingly, increase in the proportion of cells in G2/M phase was accompanied by decrease in cell population of G1 phase (Fig. 2a–d). Similar results were obtained in HeLa cells (Fig. S2a**)** however; MDA-MB-231 cells (Fig. S2b) showed significant but comparatively lower G2/M arrest. We also observed significant time-dependent increase in phospho-histone H3(Ser10) indicative of mitotic cells with condensed DNA^15^, in presence of 10 µM of Z-DAN-11 (Fig. 2e,f and Fig. S2c). However, MCF-7 and A549 cells showed relatively higher pH3-ser10-positive cells as compared to MDA-MB-231 which correlated with propidium iodide staining. Collectively these results suggest that Z-DAN-11 treatment induce G2/M arrest thereby playing a critical role in blocking cellular proliferation.

### Z-DAN-11 induces differential expression of distinct gene sets involved in cell cycle and cell death-related processes

**Fig. 3:**
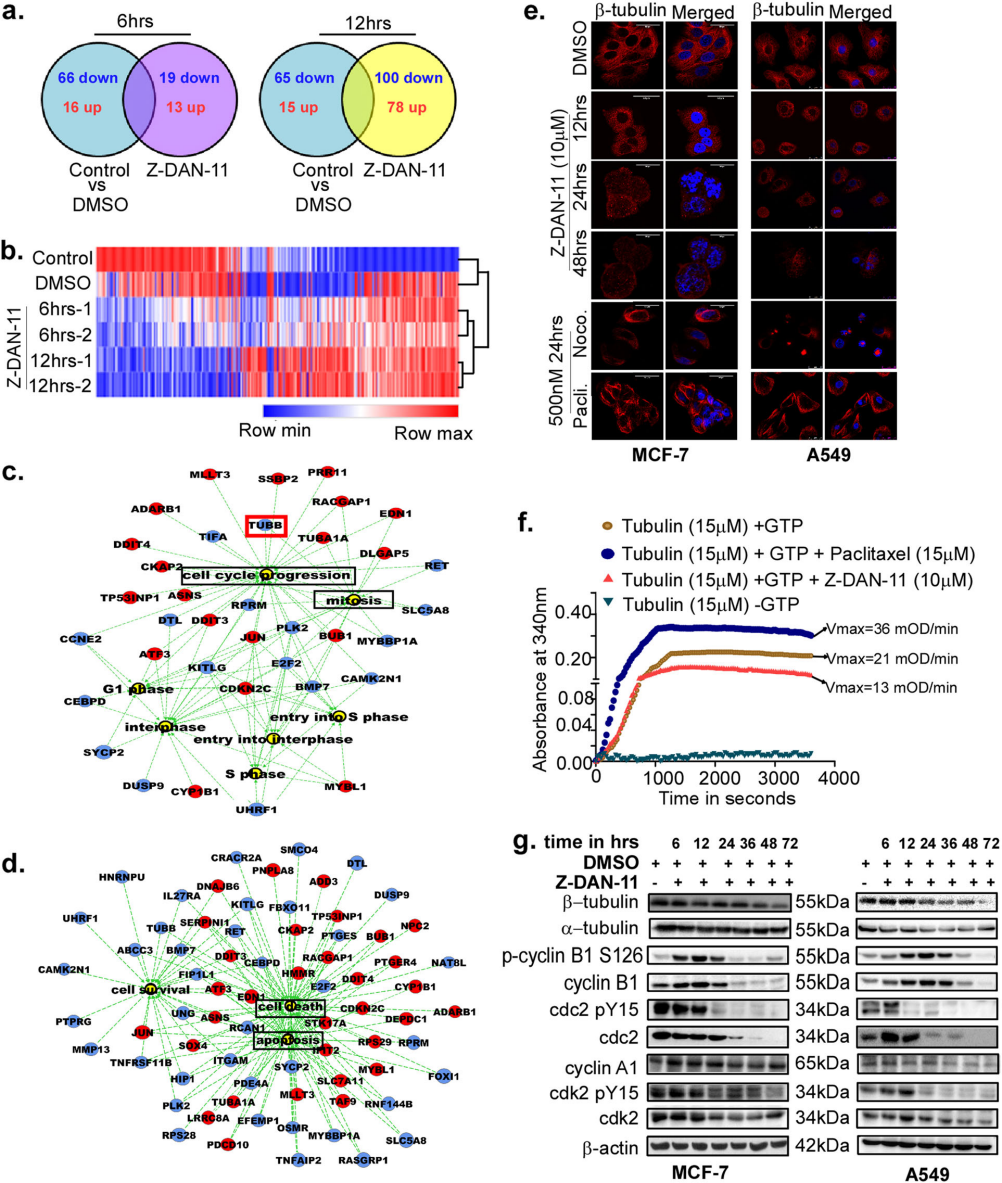
Z-DAN-11 induces differential expression of distinct gene sets involved in cell cycle and cell death-related processes. **a** Microarray analysis representing differentially regulated genes (DRGs) in Z-DAN-11 treated MCF-7 cells at 6 and 12 h compared to DMSO control and untreated cells (*p* ≤ 0.05, FC ≥ 2). **b** Heat map showing distinct contrast in gene expression between the Z-DAN-11 treated and the control samples. Gene interaction network involving DRGs specific to Z-DAN-11 treated cells at 12 h time point and involved in **c** cell cycle and **d** cell death-related functions. Upregulated genes are marked in red, downregulated genes are marked in blue and functions are marked in yellow. **e** Photomicrographs showing time-dependent decrease in β-tubulin in MCF-7 and A549 cells in response to Z-DAN-11 treatment. Briefly, cells were treated with Z-DAN-11 (10 μM) for the indicated time points and fixed with 3.7% formaldehyde. Post fixation cells were permeabilized with triton-X and incubated with β-tubulin antibody. Alexa-Fluor 594-conjugated secondary antibody was used followed by counter staining with DAPI. Finally, images were acquired in Leica confocal microscope (magnification: ×63). **f** Effect of Z-DAN-11 on in vitro tubulin polymerization. Purified porcine tubulin was incubated with or without Z-DAN-11 or paclitaxel in tubulin polymerization buffer and kinetic loop study was carried out. **g** Western immunoblot data depicts Z-DAN-11-mediated alternation in the expression of cell cycle-related proteins including α-tubulin and β-tubulin, cyclin A1, cyclin B1, phospho-cyclin B1 (S126), cdc2, phospho-cdc2 (Tyr15), cdk2, and phospho-cdk2 (Tyr15) in MCF-7 (left panel) and A549 cells (right panel)

In order to observe the systemic impact of Z-DAN-11 treatment in gene expression, we did transcriptome analysis of Z-DAN-11 treated MCF-7 cells at 6 and 12 h, along with untreated and DMSO treated cells using microarray. Comparative analysis revealed that Z-DAN-11 treatment led to time-dependent increase in the number of differentially regulated genes (DRGs), however the overall numbers of DRGs were highest at 12 h (Fig. 3a). Hierarchical clustering of the cell samples based on their gene expression, along with a heat map, demonstrate a distinct differential gene expression signature among the Z-DAN-11 treated and control samples (Fig. 3b). Functional enrichment analysis of the DRGs specific to the Z-DAN-11 treated cancer cells using IPA showed significant association of these genes (Tubb, Tuba, c-Jun, Tp53, Ddit3, Tgfbi etc.) in cell cycle and cell death-related processes (Fig. 3c,d), corroborating with our experimental validations (Fig. S3a-b).

### Z-DAN-11-mediated disruption of micro-tubular dynamics leads to G2/M cell cycle arrest

Microarray data revealed altered expression of several genes involved in cell cycle regulation as well as cell death-related processes in response to Z-DAN-11 in MCF-7 cells, including but not limited to β-tubulin, which were validated by q-PCR (Fig. S3a-b). Both microarray as well as RT-PCR demonstrated comparable fold changes in the expression of these genes (Fig. S3b). Our western immunoblot and immunofluorescence data showed reduced expression of β-tubulin (Fig. 3e,g) but not α-tubulin (Fig. 3g and Fig. S2d), which play critical role during the M-phase of cell division process^16^. We observed microtubule networks in DMSO as well as paclitaxel (a drug known to stabilize tubulin polymers^17,18^) treated condition in MCF-7 and A549 cells. On the contrary, there was a time-dependent decrease in the expression of β-tubulin indicative of disrupted microtubule networks in Z-DAN-11 (10 μM) treated cells similar to those treated with the drug, nocodazole^19^ (Fig. 3e). Similar results were obtained with MDA-MB-231 cells (Fig. S2e). Further, confirmation of tubulin depolymerization by Z-DAN-11 was obtained from kinetic loop experiment with purified porcine tubulin, which shows that Z-DAN-11-mediated (*V*_max_ = 13 mOD/min) tubulin polymerization was comparatively lower than the control condition (*V*_max_ = 21 mOD/min) while with paclitaxel (*V*_max_ = 36 mOD/min.), polymerization efficiency was much higher (Fig. 3f). These results indicate that Z-DAN-11 interferes with microtubule polymerization process which leads to G2/M arrest. Interestingly Schneider et al. discussed inhibition of tubulin polymerization for other Resveratrol derivative which is structurally different from Z-DAN-11 but alike Z-DAN-11 it causes G2/M arrest through inhibition of tubulin polymerization^20^. Moreover, western blot analysis of critical cell cycle regulatory proteins (Fig. 3g) show significant time-dependent decrease in the expression level of total Cdc2 along with phospho-cdc2 (Tyr15) in response to Z-DAN-11 treatment in both MCF-7 (Fig. 3g, left panel) and A549 cells (Fig. 3g, right panel). While cyclin B1 expression level initially increased up to 24 h, significant decrease in cyclin B1 levels were observed post 36 h treatment with the compound (Fig. 3g). Phospho-cyclin B1 (S126) also followed quite similar expression pattern as cyclin B1 both in MCF-7 and A549 cells. Cdk2 and phospho-cdk2 expression also showed a time-dependent decrease in response to Z-DAN-11 treatment, with decrease mostly evident at late time points. Again, while Z-DAN-11 treatment did not affect the expression of cyclin A1 in MCF-7, time-dependent decrease in cyclin A1 was observed in A549 after 36 h (Fig. 3g).

### Z-DAN-11 treatment induces DNA fragmentation, ROS-mediated mitochondrial permeability transition, and apoptosis in human cancer cells

**Fig. 4:**
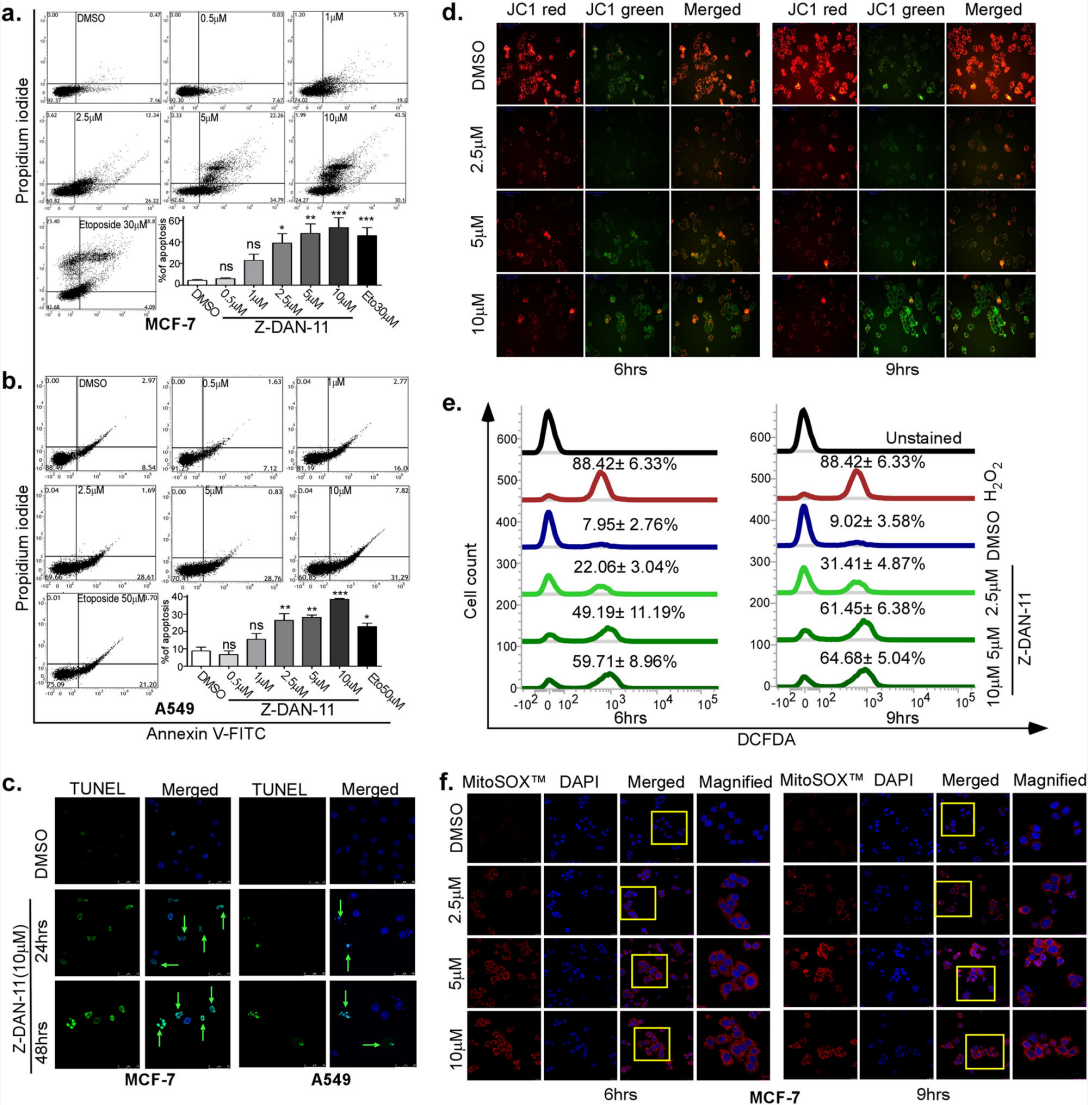
Z-DAN-11 treatment induces DNA fragmentation, ROS mediated mitochondrial permeability transition (MPT) and apoptosis in human cancer cells. **a**, **b** Concentration-dependent increase in annexin V-FITC/PI-positive population of MCF-7 and A549 cells, respectively, in response Z-DAN-11 treatment. Post treatment with Z-DAN-11(0–10 µM) for 48 h, cells were stained with annexin V-FITC/PI and analyzed in FACs. **c** Identification of apoptotic nuclei by TUNEL staining showing time-dependent increase in TUNEL positivity in MCF-7 and A549 cells (marked with arrows). Briefly, cells were treated with Z-DAN-11 (10 μM) for the indicated time points. After fixation, cells were stained with TUNEL and counterstained with DAPI for visualization of nuclei. Images were obtained in Leica confocal microscope (magnification: ×40). **d** Microscopic images of JC1 staining in MCF-7 cells indicating Z-DAN-11-induced time-dependent and concentration-dependent increase in the mitochondrial permeability which is evident from the red to green shift of fluorescence. **e** Flow cytometric data showing time-dependent and concentration-dependent increase in intracellular ROS in MCF-7 cells in response to the treatment of Z-DAN-11. Post treatment, DCFDA (5 µM final) in serum-free media was added and incubated for 30 min; cells were scrapped out and washed in 1× PBS. Finally the fluorescent signals from the cells were acquired by FACS-Verse. **f** Microscopic images of MitoSOX™ Red staining in MCF-7 cells demonstrating Z-DAN-11-induced time-dependent and concentration-dependent increase in the mitochondrial ROS as evident from increase in red fluorescence. Briefly, cells grown on coverslips were fixed with 3.7% formaldehyde, were washed with 1× PBS and DAPI staining was done. Coverslips with stained cells were mounted on slides and observed in Leica confocal microscope

**Fig. 5:**
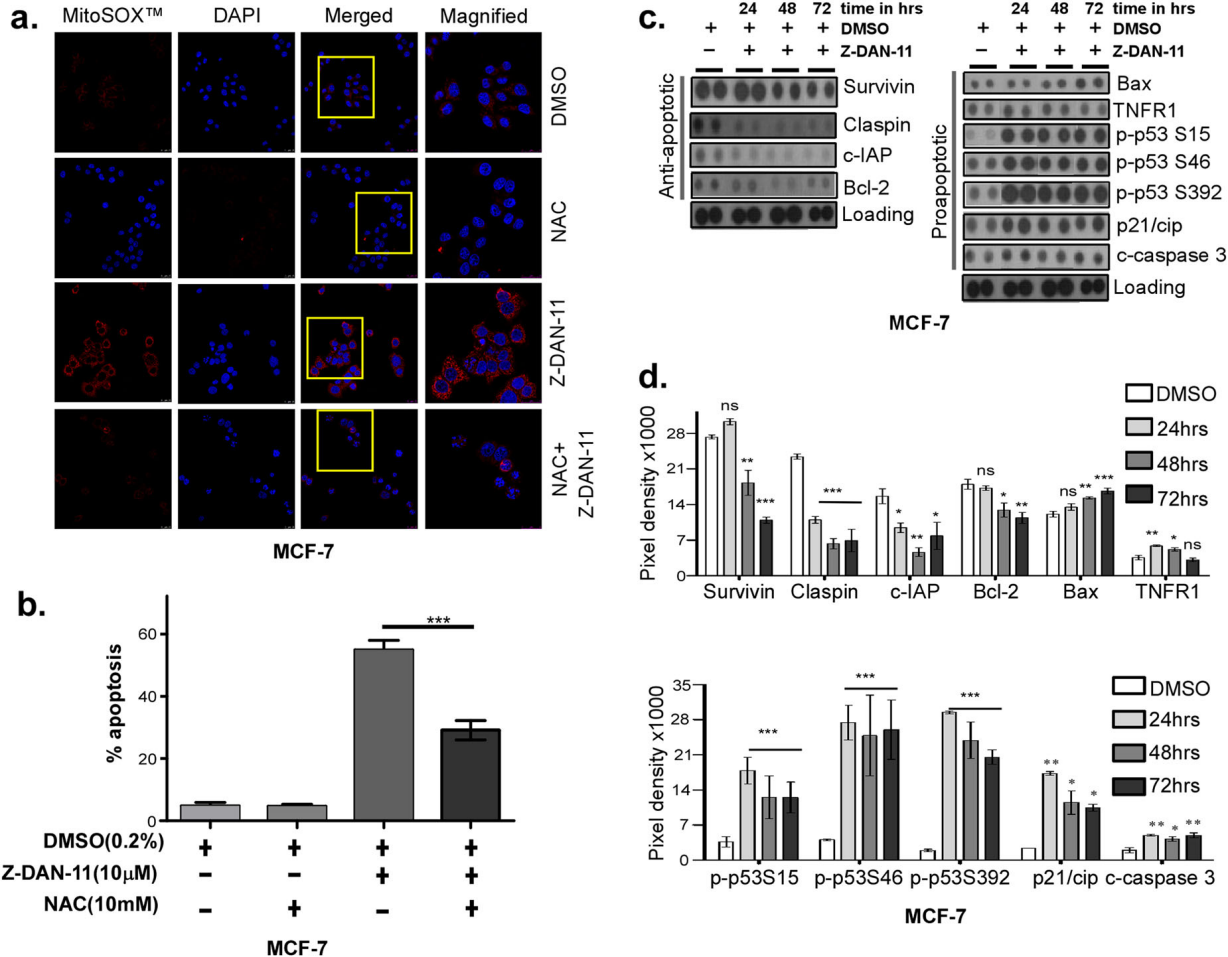
Z-DAN-11-induced ROS production contributes to mitochondrial dysfunction and cancer cell apoptosis. **a** Confocal microscopic images demonstrating protection of Z-DAN-11-mediated mitochondrial ROS production by N-acetyl-cysteine (NAC). Briefly, 5 × 10^4^ MCF-7 cells grown on coverslips were pre-treated with 10 mM ROS scavenger NAC for 4 h followed by treatment with 10 µM of Z-DAN-11 for 6 and 9 h. Cells were fixed with 3.7% formaldehyde and washed with 1× PBS. DAPI staining was done and washed with 1× PBS. Coverslips with stained cells were mounted on slides and observed in Leica confocal microscope. **b** Showing the involvement of ROS in Z-DAN-11-mediated mitochondrial dysfunction in MCF-7 cells apoptosis. Briefly, MCF-7 cells were pre-treated with 10 mM ROS scavenger NAC for 4 h followed by treatment with 10 µM of Z-DAN-11 for 48 h. Finally cells were trypsinized, stained with Annexin V-FITC/PI and analyzed by flow cytometry. **c** Human apoptosis proteome profile array demonstrates Z-DAN-11-induced differential expression of pro-apoptotic and anti-apoptotic proteins along with their **d** pixel density. Data are representative of two independent experiments and bar graph shows mean ± SEM (*p < 0.05**p < 0.01, ***p < 0.001, ns not significant)

Forty-eight hours post treatment with different concentrations of Z-DAN-11 (0–10 µM) showed dose-dependent induction of apoptosis in MCF-7, A549 as well as MDA-MB-231 cells, as indicated by the increased percentage of annexin V-FITC+ve cells (Fig. 4a,b and Fig. S4a). While the minimal concentration required for significant induction of apoptosis by Z-DAN-11 in MCF-7 and A549 cells (Fig. 4a,b) was 2.5 µM, in MDA-MB-231 cells (Fig. S4a) it was around 5 µM. Apoptosis induction in response to Z-DAN-11 was followed microscopically by transferase dUTP nick end labeling (TUNEL) assay^21^. Time-dependent increase in TUNEL-positive cells were observed in MCF-7, A549 (Fig. 4c) as well as MDA-MB-231 cells (Fig. S4b) in response to Z-DAN-11. DAPI staining also revealed profound DNA damage caused by Z-DAN-11 (Fig. S4c). Moreover, we investigated the effect of Z-DAN-11 on mitochondrial damage by JC-1 staining which showed a drastic alteration of the redox status of cellular mitochondria in response to Z-DAN-11 in a concentration-dependent and time-dependent manner both in MCF-7 and A549 cells (Fig. 4d and Fig. S5a).The shift of fluorescence from red to green or a decrease in the red/green ratio indicated the increase in the mitochondrial permeability in response to Z-DAN-11. In addition to that we also checked both cellular (Fig. 4e and Fig. S5b) and mitochondrial ROS (Fig. 4f and Fig. S5c) production in response to Z-DAN-11 which showed dose-dependent and time-dependent increase in cellular as well as mitochondrial ROS. Our observation showed that pretreatment of 10 mM N-Acetyl-Cysteine (NAC) was not only able to reduce Z-DAN-11-mediated mitochondrial ROS (Fig. 5a and Fig. S5d) but also decreased cancer cell apoptosis significantly (Fig. 5b and Fig. S5e), thereby confirming that Z-DAN-11-mediated cancer cell apoptosis is due to increased ROS production.

### Z-DAN-11 perturbs the balance in the pro-apoptotic/anti-apoptotic signaling to induce apoptosis in cancer cells

A time-dependent proteome profile was performed with MCF-7 cells treated with Z-DAN-11 using the Proteome Profiler^™^ Human Apoptosis Array Kit (R&D Bio-system), as shown in Fig. 5c,d and Fig. S4d. Proteome profiler data revealed Z-DAN-11-mediated time-dependent increase in the expression of several pro-apoptotic proteins (Fig. 5c,d) like Bax, p21/cip, and cleaved caspase 3, and decrease in a number of anti-apoptotic (survival) proteins (Fig. 5c,d) namely, Survivin, Claspin, c-IAP, and Bcl-2. Although TNFR1 expression did increase significantly at 24 h (Fig. 5c,d), interestingly it decreased to almost normal levels by 72 h. Figure 5d represents mean pixel densities of each protein from two separate proteome profile experiments. A time-dependent induction in phosphorylation of p53 was observed at ser15, ser46, and ser392 (Fig. 5c,d) in Z-DAN-11 treated cells versus the control cells, indicating a possible p53 dependency in Z-DAN-11-mediated apoptosis. Thus, these results suggest induction of an apoptotic cascade involving an imbalance between pro-apoptotic and anti-apoptotic proteins, which may be p53 dependent.

### Z-DAN-11-mediated apoptosis of cancer cells involve both intrinsic and extrinsic pathway

**Fig. 6:**
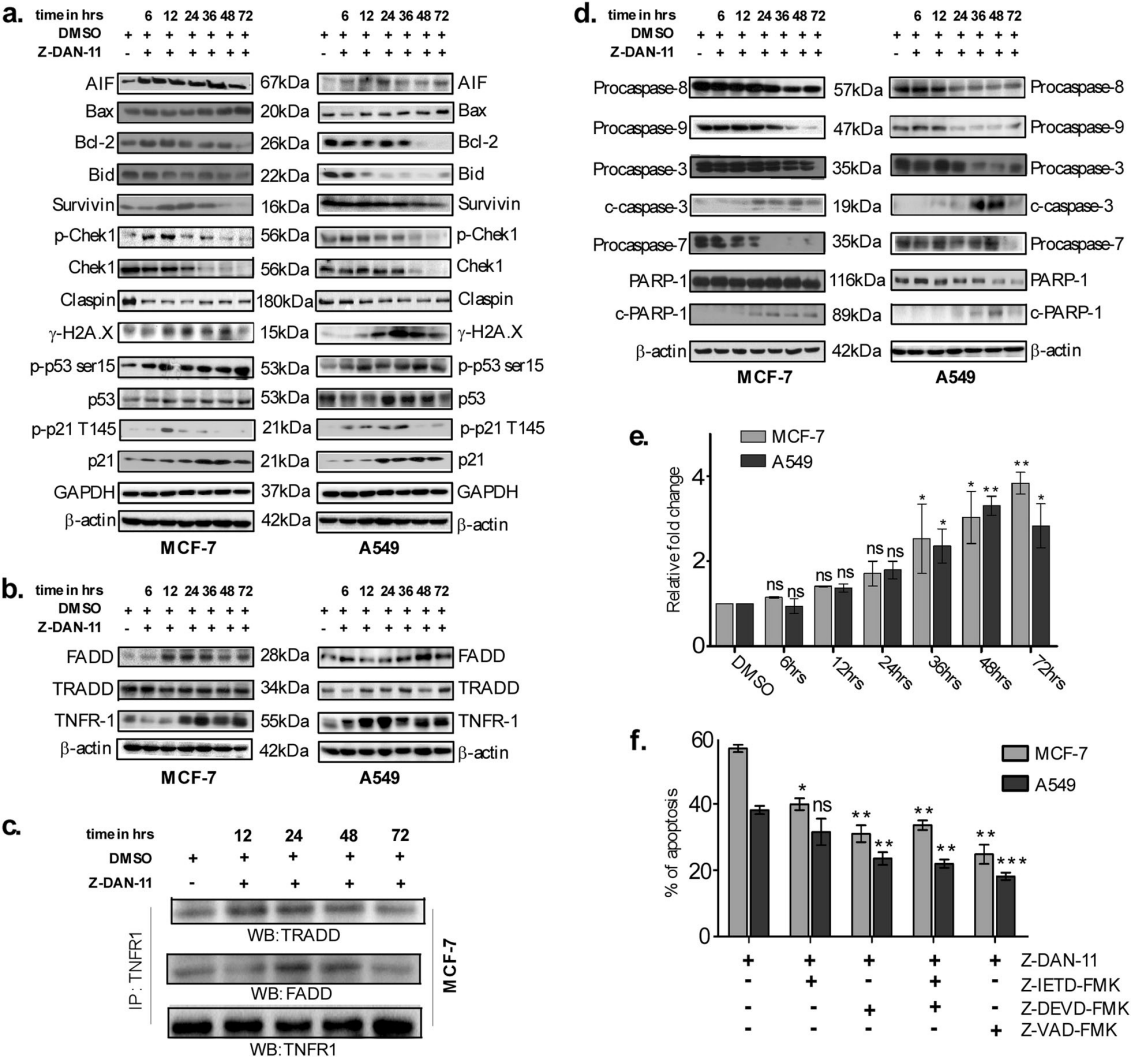
Z-DAN-11-mediated apoptosis of cancer cells involve both intrinsic and extrinsic pathway through activation of the caspase cascade leading to PARP1 cleavage. **a** Western blot images showing expression of several pro-apoptotic/anti-apoptotic proteins involved in Z-DAN-11-induced apoptosis in MCF-7 and A549 cells respectively. Briefly, cell lysates were prepared from MCF-7 and A549 cells after treating with Z-DAN-11 (10 µM) by using RIPA buffer. For immunoblotting, 20–35 µg proteins were resolved on 8–15% SDS-PAGE. Following gel electrophoresis, proteins were transferred onto PVDF membrane (Millipore, USA), blocked with 5% BSA in 1× TBST and probed with primary antibodies against several extrinsic and intrinsic pathway-related proteins and their corresponding secondary antibodies. Finally blots were developed in X-Ray films or Bio-Rad Chemidoc by ECL method. GAPDH or β-actin was used as loading controls. **b** Representation of the expression profiles of TRADD and FADD with TNFR1 in MCF-7 and A549 cells. **c** Immunoprecipitation with TNFR1 followed by western immunoblot analysis confirmed the co-immunoprecipitation of TRADD and FADD with TNFR1, indicating the formation of DISC. **d** Western blot data showing induction of Z-DAN-11-mediated apoptosis involves activation of the caspase cascade leading to PARP1 cleavage. **e** Depicts caspase 3/7 activation in response to treatment of Z-DAN-11 in a time-dependent manner in MCF-7 and A549 cells. **f** Demonstrates the involvement of caspases in Z-DAN-11-mediated apoptosis in MCF-7 and A549 cells. Briefly, MCF-7 and A549 cells were pre-treated with 20 µM each of the corresponding inhibitors for caspase 3 (Z-DEVD-FMK), caspase 8 (Z-IETD-FMK), and PAN-caspase (Z-VAD-FMK) for 4 h followed by treatment with 10 µM of Z-DAN-11 for 48 h. Finally cells were trypsinized, stained with Annexin V-FITC/PI and analyzed by flow cytometry. Data are representative of three independent experiments and bar graph shows mean ± SEM (*p < 0.05**p < 0.01, ***p < 0.001, ns not significant)

Since, mitochondrial damage (Fig. 4d and Fig. S5a) play a critical role in apoptosis signaling^22^, the effect of Z-DAN-11 on the levels of key mediators regulating mitochondrial damage were assessed. Western blot data suggest increase in cytosolic AIF (apoptosis-inducing factor) expression with time in both MCF-7 as well as A549 cells time dependently in response to Z-DAN-11 (Fig. 6a), as well as over-expression of the pro-apoptotic proteins Bax, while Bcl-2 expression was significantly lowered (Fig. 6a). Z-DAN-11 also lowered the expression of anti-apoptotic proteins Survivin and Claspin (Fig. 6a). Since, Z-DAN-11-induced DNA fragmentation (Fig. 4c and Fig. S4b-c) expression profile of critical proteins associated with maintenance of genomic integrity was studied in response to treatment with the compound. Z-DAN-11 treatment showed significant decrease in Chek1 expression time dependently (Fig. 6a). More importantly, while level of p-chek1 (ser317) was elevated at early time points, significant decrease in p-chek1 was observed at later time points (Fig. 6a). Marked lowering in expression of Claspin, a DNA-binding protein critical for phosphorylation and activation of the Chek1 protein kinase by ATR in response to DNA damage^23^, was observed in Z-DAN-11-treated conditions versus control (Fig. 6a). These data were corroborated with an elevated expression of the DNA fragmentation marker γ-H2A.X in MCF-7 as well as A549 cells (Fig. 6a). A time-dependent induction in p53 as well as p-p53 ser15 was observed (Fig. 6a) which correlated to the proteome profiler data (Fig. 5c,d).The p21/cip expression increased time dependently while level of p-p21 (Thr145) increased at early time points but significant decrease was observed at later time points. TNFR1 showed profound increase in expression in response to Z-DAN-11 in MCF-7 as well as A549 cells (Fig. 6b), which correlated with time-dependent increase in both FADD and TRADD expression, suggesting formation of the death-inducing signaling complex (DISC). Confirmation of recruitment of the DISC was obtained from Co-IP data (Fig. 6c), where TNFR1 immunoprecipitated with both TRADD as well as FADD in a time-sensitive manner, following Z-DAN-11 treatment in MCF-7 cells.

### Induction of Z-DAN-11-mediated apoptosis involves activation of the caspase cascade leading to PARP1 cleavage

The activation of caspase 3 was demonstrated by a time-dependent increase in expression of corresponding cleaved caspase 3 accompanied with decrease in their pro-form both in MCF-7 and A549 cells (Fig. 6d) following treatment with Z-DAN-11(10 μM) at several time points (6–72 h). However, in both cells the activation of caspase 7 and caspase 8 were evident from decreased expression of their pro-form after 36 h of treatment (Fig. 6d). Further, Z-DAN-11 was found to downregulate pro-caspase 9 levels time dependently, indicating activation of caspase 9 and thereby suggesting mitochondrial involvement in Z-DAN-11-mediated apoptosis. The activity assay also confirmed activation of caspase 3/7 in MCF-7 and A549 cells at later time points (36, 48, and 72 h) as shown in Fig. 6e. Additionally, cleavage mediated deactivation of PARP1 was observed in both MCF-7 as well as A549 cells (Fig. 6d). Importantly, MCF-7 and A549 pre-treated with 20 µM each of the corresponding inhibitors for caspase 3 (Z-DEVD-FMK), caspase 8 (Z-IETD-FMK), and PAN-caspase (Z-VAD-FMK) conferred significant protection to the cells from Z-DAN-11-mediated apoptosis as evidenced from Fig. 6f, confirming that Z-DAN-11-mediated tumor cell apoptosis was caspase dependent. Collectively these results suggest that Z-DAN-11 involves both the extrinsic and intrinsic pathway causing mitochondrial damage, activation of caspases leading to PARP1 cleavage, DNA damage, and eventually tumor cell apoptosis.

### Z-DAN-11-mediated apoptosis of tumor cells is p53 dependent

**Fig. 7:**
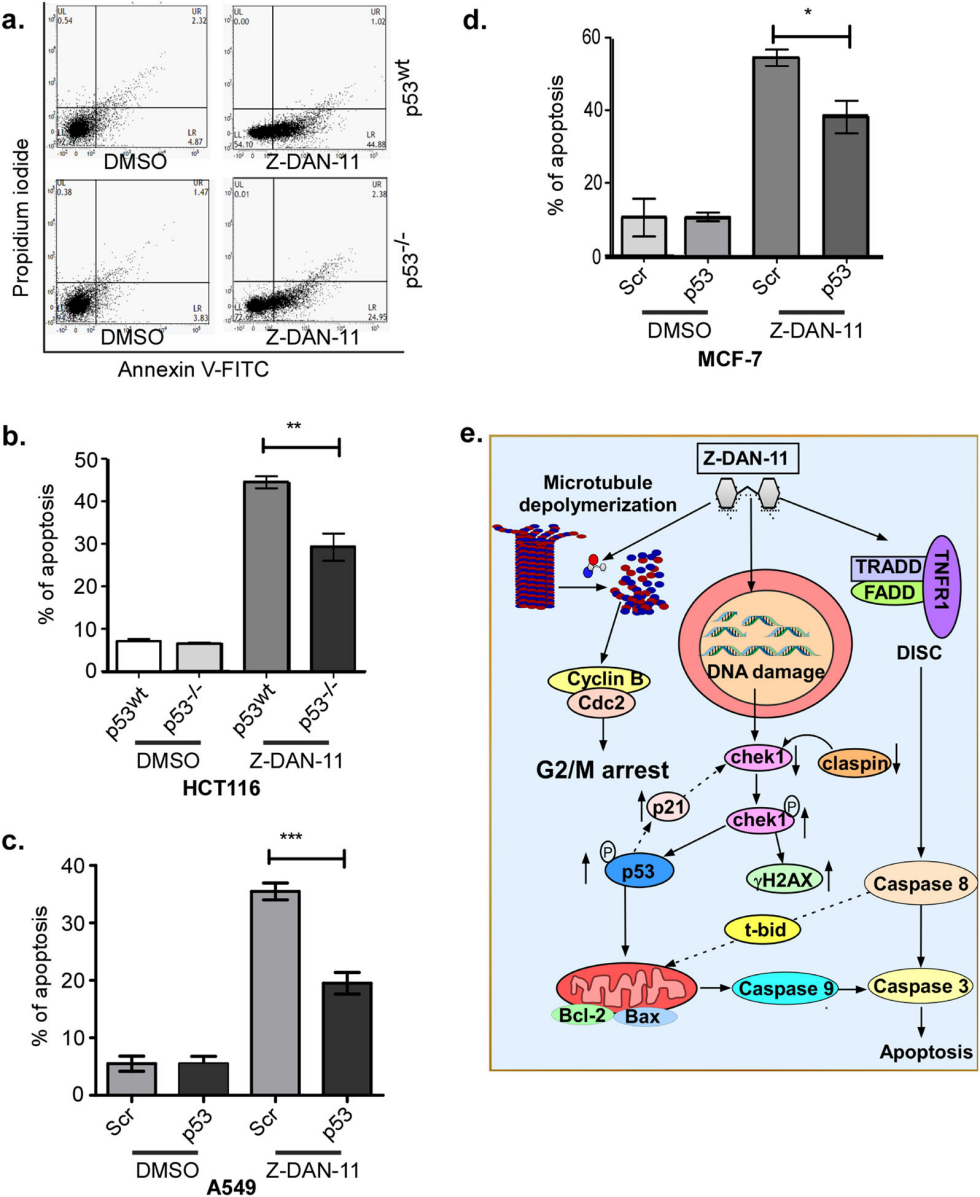
Z-DAN-11-mediated apoptosis of tumor cells is p53-dependent. **a** Depicts a representative density plot showing that Z-DAN-11-mediated apoptosis is significantly reduced in HCT116 p53^−/−^ cells compared to HCT116 p53^wt^ cells along with **b** bar graph representing mean ± SEM (*p > 0.05, **p < 0.01, ***p < 0.001, ns not significant) of at least three independent experiments. Bar diagram showing that Z-DAN-11-mediated apoptosis is blocked in p53 siRNA transfected **c** A549 and **d** MCF-7 cells compared to the scrambled. Data are represented as mean ± SEM (*p > 0.05, **p < 0.01, ***p < 0.001, ns not significant). **e** A schematic signaling model proposing how Z-DAN-11-mediated proliferation inhibition is associated with G2/M arrest and apoptosis. Z-DAN-11 binds to tubulin and induces microtubule depolymerization which leads to G2/M arrest. On the other hand Z-DAN-11-induced DNA damage contribute to apoptosis through intrinsic and extrinsic pathways involving chek1, p53, TNFR1, TRADD, FADD etc. and downstream activation of caspase 8, caspase 9, and caspase 3 leading to apoptosis

Since, proteome profiler followed by western blot analysis indicated time-dependent phosphorylation of p53 (Fig. 5c,d and Fig. 6a), we wanted to confirm the role of p53 in Z-DAN-11-mediated apoptosis of tumor cells. While in p53^wt^ HCT116 cells, Z-DAN-11 caused a significant induction of apoptosis (>40% Annexin V+ve cells shown in Fig. 7a,b), in the p53^−/−^ HCT116 cells it is significantly lower (around 27% Annexin V+ve cells), thereby confirming that p53 plays a definitive role in Z-DAN-11-mediated death of tumor cells. This was further confirmed by silencing p53 levels in both A549 and MCF-7 cell line using specific siRNAs targeted against p53, which conferred significant protection from Z-DAN-11-mediated apoptosis, in A549 (nearly 50% lower than the scrambled, Fig. 7c) and MCF-7 (nearly 35% lower, Fig. 7d) p53-siRNA-transfected cells. Together, these experiments confirmed that Z-DAN-11-mediated tumor cell apoptosis is at least partly but significantly p53-dependent. Taken together, these results suggest Z-DAN-11 depolymerizes microtubules leading to G2/M arrest, and at the same time induce DNA damage, leading to p53-dependent induction of the apoptotic cascade that involves both the intrinsic as well as the extrinsic pathway (Fig. 7e).

### Z-DAN-11 caused significant reduction in tumor growth and progression in immuno-competent mice

**Fig. 8:**
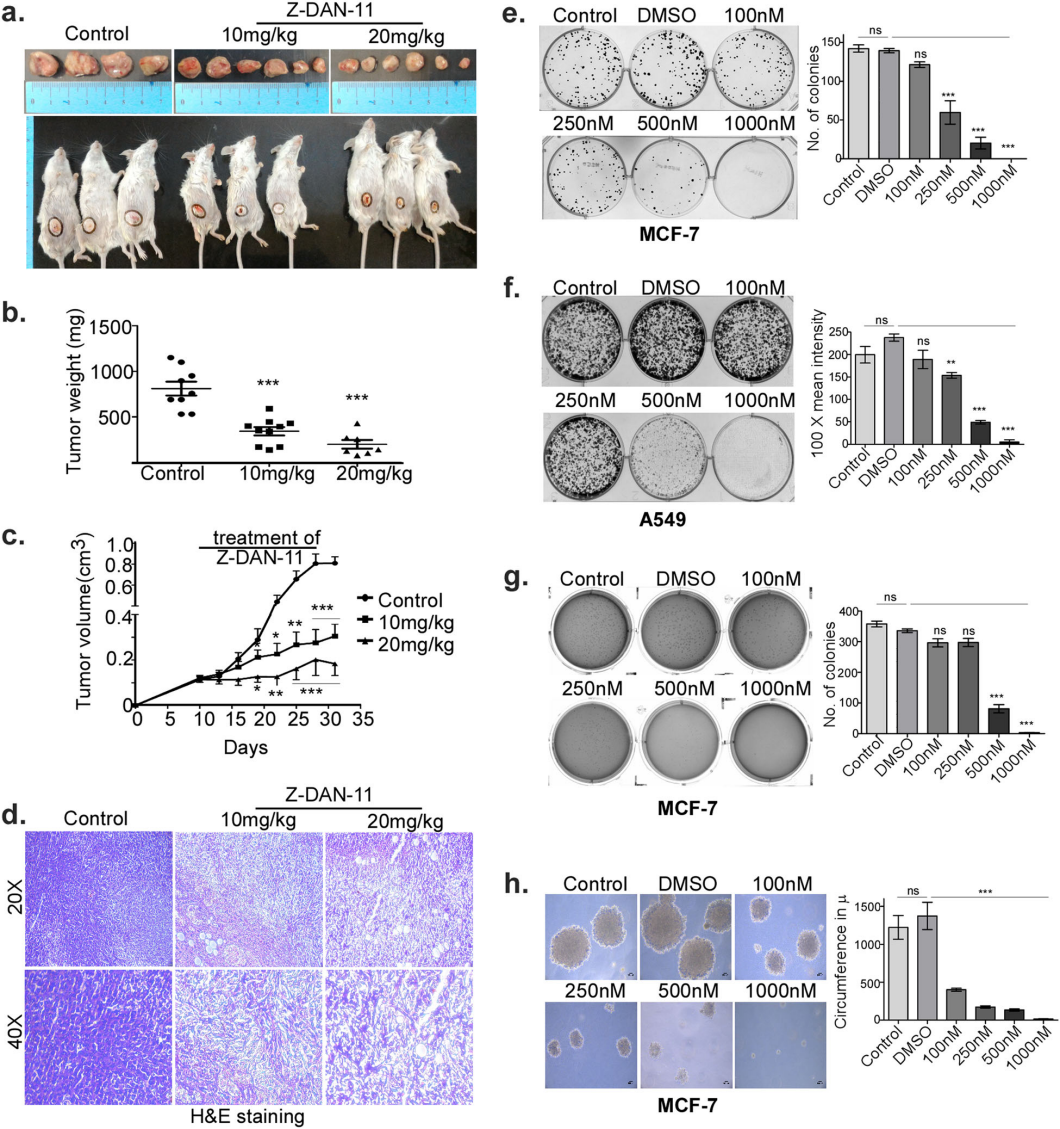
Z-DAN-11 reduced anchorage-independent growth (AIG) potential and clonogenicity of cancer cells, and demonstrates anti-tumor activity in vivo. **a** Images of resected tumors along with mice bearing tumors obtained after killing. **b** The weight of resected tumors showing significant reduction of tumor weight in Z-DAN-11 treated condition (*n* ≥ 6). **c** Represents the 4T1 tumor growth curves indicating dose-dependent and time-dependent regression of tumor volume in response to intraperitoneal injection Z-DAN-11. **d** Histological images of tumors sections with H&E staining (magnification: ×20 and ×40) showing loosely arranged cells in treated conditions in contrast to compact arrangement of cells in control tumor section. **e**, **f** Represent dose-dependent inhibition of clonogenicity in MCF-7 and A549 cells respectively. **g** Data demonstrating Z-DAN-11-mediated reduction of anchorage-independent growth (AIG) of MCF-7 cells. **h** Photomicrographs showing individual colonies indicating concentration-dependent inhibitory effect of Z-DAN-11 on AIG. Data are representative of three independent experiments and bar graph shows mean ± SEM (*p < 0.05, **p < 0.01, ***p < 0.001, ns not significant)

In vivo data shows a significant dose-dependent inhibition of tumor growth as evidenced from the reduction in tumor volume as shown in Fig. 8a, demonstrating that mice treated with either 10 mg/kg or 20 mg/kg b.w. of Z-DAN-11 have significantly smaller tumor size than the non-treated mice. This is also reflected in the weight of resected tumors from mice treated with Z-DAN-11, which shows dose-dependent decrease in the tumor weight compared to the non-treated mice (Fig. 8b). Z-DAN-11 also caused a dose-dependent decrease in tumor progression with time, as shown in Fig. 8c. Microscopic analysis of tumor section with H and E staining showed compact arrangement of cells in control tumor section in contrast to loosely arranged cells in treated conditions. Additionally, more eosin-stained cells were observed in treated condition as compared to control tumor (Fig. 8d). However, there seems to be no significant change in total body weight of mice or the serum levels of alanine transaminase (ALT), aspartate transaminase (AST), creatinine, urea and alkaline phosphatase (ALP), either treated or non-treated, as shown in Fig. S6a. The pharmacokinetics of Z-DAN-11 showed 11.23 ± 0.68% of absolute bioavailability (Fig. S6b).

### Z-DAN-11 reduced anchorage-independent growth (AIG) potential and clonogenicity of cancer cells

Z-DAN-11 significantly inhibits colony forming ability of the tumor cells at concentrations as low as 250 nM in case of MCF-7 (Fig. 8e) and 500 nM in case of A549 cells (Fig. 8f), with a complete abrogation of colony forming ability of both cells at 1 µM dose of Z-DAN-11 (Fig. 8e,f). Moreover, Z-DAN-11 exhibited inhibition of anchorage-independent growth (AIG) of tumor cells at 500 nM and above (Fig. 8g); indicating Z-DAN-11 treatment results in a loss of ability of the tumor cells to grow anchorage independently. A dose-dependent reduction in the size of individual colonies was evident from the photomicrographs shown in Fig. 8h.

## Discussion

One of the major obstacles of cancer chemo-prevention is undesirable toxicity to the normal cells, thereby resulting in adverse side-effects. This study was aimed in identification of a possible candidate with potent chemotherapeutic potential and minimal toxicity. Here, we synthesized a series of *trans-*stilbene derivatives and identified at least one derivative, named Z-DAN-11 which blocked cellular proliferation selectively in a series of different cancer cell lines in vitro, concentration dependently (Fig.1a–e and Table S1).

Functionally, Z-DAN-11-arrested tumor cells in the G2/M-phase both time and dose dependently (Fig. 2a–d and Fig. S2a-b). Z-DAN-11 caused a time-dependent decrease in cdc2, cdk2 and their phosphorylation, which are classical molecular signatures (Fig. 3g) leading to progression through the G2/M-phase, thereby confirming that Z-DAN-11 induces G2/M-phase arrest. Again as microtubule dynamics is essential for cell cycle regulation, perturbation of which triggers cell cycle arrest and apoptosis^24–27^. Mechanistically, Z-DAN-11 caused a significant downregulation of β-tubulin (Fig. 3e and Fig. S2e) similar to nocodazole. These results indicate that Z-DAN-11 drives G2/M arrest through microtubule depolymerization which may provide a possible strategy to reverse cell cycle-mediated drug resistance through synchronization of cells^28^.

Moreover, induction of cell cycle arrest was accompanied by a parallel induction of apoptosis, as evidenced from a dose-dependent increase in the number of annexin V/PI and TUNEL positive cells (Fig. 4a–c and Fig. S4a-b). Our experimental evidences (Fig. 4d–f and Fig. S5a-c) confirmed role of Z-DAN-11-mediated ROS generation in dramatic alteration of the mitochondrial redox status that critically contribute to apoptosis, as NAC was able to reduce Z-DAN-11-mediated apoptosis significantly (Fig. 5a,b and Fig. S5d-e). These data clearly suggests that Z-DAN-11-induced ROS production contributes to mitochondrial dysfunction which leads to the cancer cell apoptosis through involvement of both, intrinsic as well as extrinsic pathways. This was confirmed from increase in expression of mediators (Fig. 5c,d and Fig. 6a-d**)** that leads to mitochondrial damage (Fig. 4d and Fig. S5a), activation of caspases (Fig. 6d,e) and PARP1 cleavage.

Our findings (Fig. 6a) showed time-dependent decrease in expression of claspin along with a significant decrease in the expression of total chek1 and p-chek1 (ser317) in response to Z-DAN-11 treatment. This was associated with an elevated expression of γ-H2A.X (Fig. 6a), which may provide a possible link between the observed apoptosis and G2/M-phase arrest mediated by Z-DAN-11 treatment. Further, since inhibition of chek1 was known to cause G2 abrogation thereby selectively promoting apoptosis in cancer cells but not in normal cells^29^, Z-DAN-11-mediated chek1 inhibition may also explain the selective cytotoxicity of the compound observed earlier. Thus, chek1 plays a critical role in the observed G2/M arrest, DNA fragmentation and subsequent Z-DAN-11-induced apoptosis. As p53 phosphorylation at ser15 (DNA damage response)^30,31^, ser46 (induction of apoptosis)^32,33^ and ser392 (growth suppression)^34^ play important role in cell proliferation. Interestingly, from proteome profiler and western immunoblot data, we found increased levels of total p53 along with p-p53 ser15, ser46, ser392 as shown in Fig. 5c,d and Fig. 6a, suggesting a critical role of p53 in Z-DAN-11-mediated apoptosis. Significant reduction of Z-DAN-11-mediated apoptosis both in p53^−/−^ cells (Fig. 7a,b) as well as siRNA-mediated p53-silenced A549 and MCF-7 cells (Fig. 7c,d), confirmed the involvement of p53 in Z-DAN-11-mediated apoptosis. Finally, to establish Z-DAN-11 as an effective anti-cancer lead, we investigated its therapeutic efficacy in a syngeneic mouse tumor model, using 4T1 cells on a Balb/c background. Z-DAN-11-regressed tumor volume as well as tumor weight (Fig. 8a–c) without significant adverse effect on animals (Fig. S6a). Thus, we have identified a novel synthetic *trans-*stilbene resveratrol derivative with potent anti-tumor activity, the mechanism behind which is its ability to selectively block tumor cell proliferation and induce tumor cell apoptosis, but not normal cells. Hence, Z-DAN-11 may be a promising candidate for targeted and selective therapeutic strategy against cancer.

## Materials and methods

### Chemicals and reagents

Chemicals and reagents were obtained from Sigma-Aldrich (USA), Merck (India), Invitrogen (India), Himedia (India), and SRL (India). Primers and DCFDA (#D6883) were purchased from Sigma (India). p53 si-RNA (#sc29435) and purified porcine tubulin (#T240) was bought from Santa Cruz Biotechnologies (USA) and Cytoskeleton (USA), respectively. MitoSOX™ (#M36008) was procured from Thermo Fisher scientific (USA). Various primary antibodies α-tubulin (#2125), β-tubulin (2146), Cdc2 (#9116), phospho-Cdc2 (#9111), Cdk2 (#2546), cyclin B1 (#4138), p-H3 ser10 (#9706), AIF (#5318), TRADD (#3994), FADD (#2782), TNFRI (#3736), Bax (#3994), survivin (#2808),γ-H2A.X(#80312), p53 (#2524), p-p53 (#9286), p21 (#2947), cleaved caspase 3 (#9664), caspase 7 (#9494), caspase 8 (#9746), caspase 9 (#9508), Bid (#2002), and PARP1 (#9532) were obtained from Cell Signaling Technologies, USA. Chek1 (#AF1630), p-chek1 (#AF2054), claspin (#MAB3310) antibodies, and human apoptosis proteome profiler kit (#ARY009) were purchased from R & D Bio-systems. Cyclin A1 (#sc56301), phospho-p21 (#sc-377569) and Bcl-2(#sc7382) were brought from Santa Cruz Biotechnologies (USA). Loading controls GAPDH and β-actin were procured from Imgenex (India). Cdk2 (#ab6433), phospho-Cdk2 (#ab76146), phospho-Cyclin B1 (#ab133439), caspase 3 colorimetric assay kit (#ab39401) was obtained from Abcam (USA). Inhibitors to caspase 3 (Z-DEVD-FMK #sc3075), caspase 8 (Z-IETD-FMK #sc3084), and Pan-caspase (Z-VAD-FMK #sc3067) were ordered from Santa Cruz Biotechnologies (USA). Fetal bovine serum (#16000044) was bought from Gibco, USA and l-glutamine, Gentamicin, MEM sodium pyruvate; MEM non-essential amino acids were procured from Hi-Media, India.

### Cell culture and maintenance

MCF-7, MDA-MB-231, MDA-MB-468, T47D, HBL-100 (human breast cancer cells), HeLa (human cervical cancer cells), WI-38 (human lung fibroblast cells), A549 (human lung adenocarcinoma cells), HCT116 p53^wt^, HCT116 p53^**−**/^^−^ (human colon carcinoma cells), PC3 (prostate cancer) and 4T1 (murine mammary carcinoma) cell lines were procured from the central cell repository of National Center for Cell Science (NCCS), Pune, India and cultured as suggested by the supplier. Human renal cell carcinoma (RCC) cell lines SK-RC-45 and NKE cell were obtained from Dr. Gerd Ritter (Ludwig Institute of Cancer Research, USA) and Dr. James H. Finke (Cleveland Clinic Foundation, USA) respectively. All the above cell lines were cultured either in RPMI 1640 or DMEM, containing 10% FBS, 1 mM sodium pyruvate, 2 mM l-glutamine, non-essential amino acids, 100 units/L penicillin, 100 mg/L streptomycin, and 50 mg/L gentamycin sulfate at 37 °C with 5% CO_2_.

### Synthesis of *trans-*stilbene compounds

Briefly, to an ice-cold solution of aryl acetonitriles (1–4) (1 mmol) and aldehydes (5–19) (1.1 mmol) in EtOH (10 mL) was added 5% aq. NaOH (0.2 mL) and the reaction mixture was briskly stirred at room temperature for 30 min. Acidification of the reaction mixture using 1 N HCl led to the formation of products as solids. Filtration of the solid products and re-crystallization from EtOH furnished pure products Z-DAN-1−Z-DAN-22.

### MTT cell proliferation assay

In order to determine the effect of the synthesized compounds on cell proliferation, MTT assay was performed^35,36^. Briefly, cells were grown in 96 well plate in presence of the synthesized (Z) -2, 3-diarylacrylonitrile (*trans-*stilbene) derivatives (0–60 µM) for 48 h. 5% FBS-containing phenol red-free DMEM and MTT (200 µL; 0.5 mg/mL) were added to each well and incubated at 37 °C for 4 h in a humidified incubator containing 5% CO_2_. The purple-colored formazan crystals formed in the wells were dissolved in DMSO and absorbance was measured at 570 nm with a microplate reader.

### Trypan blue exclusion method

Trypan blue exclusion is technique which classifies viable and nonviable cells based on membrane integrity. A viable cell with intact membrane will not take trypan blue whereas a nonviable cell will have trypan blue positive cytoplasm^37^. Post treatment, 1× PBS resuspended cells were mixed with 0.4% trypan blue solution in 1:1 dilution for 4 min and counted with hemocytometer.

### Cell cycle profiling assay by propidium iodide staining

For this experiment, double thymidine blocked^38^ synchronized human cancer cells including MCF-7, MDA-MB-231, A549, and HeLa were treated with increasing concentrations (0.5 µM, 1 µM, 2.5 µM, 5 µM, and 10 µM) of Z-DAN-11 for 24 h. Nocodazole was used as a reference control for this experiment^39,40^. Post treatment, cells were harvested into single cell suspension and fixed by incubating the cells overnight at −20 °C with 75% ethanol. Cells were centrifuged and resuspended in 1× PBS for 2 h followed by RNaseA (20 µm) treatment for 2 h at 37 °C. Finally, propidium iodide was added and incubated for 20 min at room temperature. Flow cytometric analysis was immediately performed using a FACS-Verse instrument (BD).

### Microarray

Affymetrix PrimeView™ Human Gene Expression Array platform was used to check the differential expression of gene sets in response to Z-DAN-11. Microarray data sets were analyzed using TM4:Microarray Software suite^41^. Gene-level signal estimates were derived from the raw data files. The ‘affy’ package in R were used to quantile normalize and background adjust the raw CEL files by implementing the Robust Multichip Averaging algorithm. SAM analysis was performed to determine the statistically significant differentially expressed genes between the test and the control groups, using default statistical parameters. Genes with FDR-adjusted *p* values ≤ 0.05 were further screened to get the final set of DRGs based on fold change ≥2 between test and the control groups. Hierarchical clustering was performed by complete linkage and Pearson’s correlation using Cluster 3.0^42^; results were visualized using Java TreeView (http://jtreeview.sourceforge.net/).

### RNA isolation and real-time PCR

Total RNA was extracted using Trizol reagent (Invitrogen). cDNA was prepared from 1 µg of RNA extracted from MCF-7 cells treated with 10 µM dose of Z-DAN-11 for 6, 12, and 24 h along with DMSO-treated control using Verso cDNA synthesis kit (Thermo Scientific). RT-PCR was performed using SYBR green PCR system on 7500 fast (Applied Biosystem). All mRNA quantification data were normalized to β-actin. Real-time primers were listed in Figure S3c.

### Immunocytochemistry

Immunocytochemistry was performed as described previously^43^. Briefly, 5 × 10^3^ cells grown on coverslips were fixed with 3.7% paraformaldehyde, washed with 1× PBS and stained with primary antibody (1:100) overnight at 4 °C. Cells were washed with 1× PBS, counterstained with Alexaflour-488/594 secondary antibody (1:500) for 1 h at room temperature. Finally, DAPI staining was done and washed with 1× PBS. Coverslips with stained cells were mounted on slides and observed in Leica confocal microscope.

### In vitro microtubule polymerization assay

Purified porcine tubulin (15 μM) was incubated with or without the compounds in tubulin polymerization buffer PEM (80 mM PIPES, 0.5 mM MgCl2, 1 mM EGTA, pH 6.8) with 10% DMSO in ice for 10 min. Subsequently, 1 mM (GTP) was added and kinetic loop study was done using Thermo scientific Multiscan Go Multi Plate reader set at 37 °C temperature. The polymerization was monitored over 60 min by measuring the absorbance at 340 nm.

### Annexin V-FITC/PI staining

Induction of apoptosis was measured by flow cytomety after annexinV-FITC/PI staining^44^ using BD Bioscience kit. Briefly, post treatment 0.5 × 10^6^ cells were washed in ice-cold 1× PBS and resuspended in 100 µL of binding buffer and incubated with 5 µL of annexin V-FITC and 5 µL of PI for 15 min at room temperature in a dark place as per manufacturer’s guidelines. Flow cytometric analysis was immediately performed using a FACS-Verse instrument (BD).

### JC1 staining

Assessment of mitochondrial permeability was measured by JC1 staining as described earlier^45^. MCF-7 cells were treated with test compounds (0, 2.5, 5, and 10 μM) for 6 and 9 h. Cells were washed with 1× PBS buffer and incubated with the JC-1 dye (3 μM final concentration in DMEM media) at 37 °C for 30 min in the dark. Cells were again washed twice with 1× PBS buffer and kept back in 1× PBS buffer. Finally images were captured with Leica fluorescent microscope.

### Measurement of mitochondrial ROS by using MitoSOX™

MitoSOX™ Red mitochondrial superoxide indicator is a novel fluorogenic dye for highly selective detection of superoxide in the mitochondria of live cells^46^. For this experiment briefly, 5 × 10^4^ cells grown on coverslips were fixed with 3.7% formaldehyde, washed with 1× PBS and DAPI staining was done. Coverslips with stained cells were mounted on slides and observed in Leica fluorescence microscope.

### Measurement of cellular ROS using DCFDA

Intracellular ROS was measured using DCFDA method^47^. MCF-7 and A549 cells were treated with test compounds (0, 2.5, 5, and 10 μM) for 6 and 9 h. After treatment, the media was discarded and serum-free media was added. Then DCFDA (5 µM final) was added and incubated for 30 min. Post incubation the media was discarded and adherent cells were scrapped out and washed in 1× PBS. Finally, the fluorescent signals from the cells were acquired by FACS-Verse.

### Human apoptosis proteome profiler array

Expression pattern of several pro-apoptotic and anti-apoptotic proteins were examined in Z-DAN-11 (10 µM) treated and DMSO-treated MCF-7 cells for 24, 48, and 72 h by using Human apoptosis array kit (R&D Biosystem). An aliquot of 300 µg of protein was used for each condition and experiment was as performed as described in our earlier report^48^. Thereafter, cell lysates were subjected to analysis using the Proteome Profiler^TM^ human apoptosis antibody array according to the manufacturer’s instructions. Arrays were developed with streptavidin-HRP for 30 min on a rocking platform shaker. Developed signals were densitized using ImageJ software, pixel densities were normalized to untreated sample and expressed as mean pixel density.

### TUNEL assay

Visual confirmation of apoptosis was achieved by using Terminal deoxynucleotidyl TUNEL assay^45^. For this experiment, 4 × 10^4^ cells were grown to confluence with or without Z-DAN-11 (10 µM) on coverslips, following which cells were washed with 1× PBS and fixed with pre-warmed 3.7% formaldehyde. Cells were then permeabilized with 0.1% triton-X at 2–8 °C for 5 min. TUNEL reaction mixture was added to the cells and incubated for 1 h in the dark in humidified chamber. DAPI was used as counter stain. Microscopic analysis was done using a confocal microscope.

### Western immunoblotting

Briefly, cell lysates were prepared from MCF-7 and A549 cells post treatment with Z-DAN-11 (10 µM) with RIPA buffer method. For immunoblotting, 20–35 µg proteins were resolved on 8–15% SDS-PAGE, transferred onto PVDF membrane (Millipore, USA), blocked with 5% BSA in 1× TBST, and probed with respective primary antibodies followed by corresponding secondary antibodies. Finally blots were developed into X-Ray films by ECL method or by using Chemidoc MP system Bio-Rad.

### Caspase 3/7 activation assay

For this experiment cell lysates were prepared from MCF-7 cells after treating with Z-DAN-11 (10 µM) with RIPA buffer method. An aliquot of 150 µg of protein was used for each condition. DTT and DEVD-p-NA substrate were added according to manufactures protocol. Samples were mixed well and incubated at 37 °C for 90 min and finally OD was measured at 405 nm in Thermo Multiskan GO microplate reader.

### Co-immunoprecipitation

Co-immunoprecipitation was performed as described previously^49^. Cells were lyzed in IP lysis buffer (25 mM Tris-HCl pH 7.4, 150 mM NaCl, 1 mM EDTA, 1% NP-40, and 5% glycerol). A total of 400 μg of protein lysates were incubated with rabbit monoclonal anti-TNFRI antibody (3 μg/condition) overnight with continuous rotation. Next day, Protein A beads were added and incubated for another 4 h. Then, beads were washed three times using lysis buffer. Finally, beads were resuspended in 40 μL of 2× laemmli buffer, centrifuged at 13,000 rpm. Supernanants were collected and resolved on 12% SDS-PAGE. Finally, western immunoblot was done with FADD, TRADD, and TNFRI antibodies.

### si-RNA transfection

MCF-7 cells (1.5 × 10^5^) were grown in 6 well plates in antibiotic-free media for 24 h. Post incubation, p53 si-RNA and scrambled were transfected with RNAi Max (Invitrogen) as per manufacturer’s instructions. Cells were grown in opti-MEM for 6 h followed by incubation with complete DMEM for 24 h.

### Soft agar colony formation assay and clonogenicity

Clonogenic assay and Soft agar colony formation assay was performed as described earlier^50^. For soft agar assay, 2 × 10^4^ cells were resuspended in 1 mL 2× complete DMEM and mixed with 1 mL warmed 0.7% agarose and plated over a solidified 0.75% agarose in 1× DMEM. Following solidification of the top layer, 2 mLof DMEM was added, incubated at 37 °C for 30 days with continuous media change (with or without Z-DAN-11) in every 3rd day. Colonies were fixed, stained with 0.05% crystal violet and imaged using Gel Doc XR+ (Bio-Rad). For clonogenicity assay, cells were plated at very low density (200 cells/well in 6 well plate), grown for either 8/10 days, fixed with 3.7% formaldehyde, stained with 0.05% crystal violet, and image was captured using Gel Doc XR+ (Bio-Rad).

### 4T1 breast tumor model

BALB/c mice 6–8-week-old were obtained from Center for Translational Animal Research (CTAR), Bose Institute, Kolkata, India and were maintained as per the guidelines of the animal ethical committee in accordance with CPCSEA guidelines (IAEC Approval No. IAEC/BI/32/2015 Dt. 26/05/2015). For in vivo tumorigenic assay, 4T1 cells (1.5 × 10^6^ cells/animal) were subcutaneously injected into the mammary fat pad of Balb/c mice to develop solid tumor. Animals with solid tumor were randomly distributed into three groups each containing six animals. One group was treated with vehicle control (10% DMSO in normal saline) whereas; other two groups were given intraperitoneal injection of Z-DAN-11 (10 mg/kg and 20 mg/kg) starting after 10 days of tumor development and continued until 31 days (seven doses, 72 h interval). Tumor progression was monitored by measuring the volume of the tumor with vernier calipers on every third day. The tumor volume was calculated by using the formula *V* = 0.5 × *a* × *b*^2^, where “a” and “b” indicate major and minor diameter, respectively^51^. Histopathological examination was performed by haematoxylin and eosin staining as described previously^52^.

### Hepatotoxicity and pharmacokinetics studies

Following seven doses Z-DAN-11(10 mg/kg and 20 mg/kg) each with 3 days interval 100 μL blood sample were taken manually into heparinized capillary tubes by piercing the saphaneous vein with a needle and subsequently into 0.5 mL microcentrifuge tubes. All blood samples were processed for plasma isolation by centrifugation at 1640 rpm for 5 min at 4 °C within half an hour of collection. Finally serum profile of ALT, AST, ALP, creatinine, urea nitrogen was checked using respective kit as per manufacture’s instruction.

Time course (predose and 0.25, 0.50, 1, 2, 4, 8, 12, 24 h) change in mean plasma concentration of Z-DAN-11 after intravenous (1 mg/kg) and oral (10 mg/kg) administration of Z-DAN-11 was done by isolating plasma samples at each time point post oral and intravenous dose. An aliquot of 100 μL blood sample were taken manually into heparinized capillary tubes by piercing the saphaneous vein with a needle and subsequently into 0.5 mL microcentrifuge tubes. All blood samples were processed for plasma isolation by centrifugation at 1640 rpm for 5 min at 4 °C within half an hour of collection. Finally, samples were analyzed using Sciex 5500 Q-Trap LC-MS/MS and agilent 1290 series high performance liquid chromatograph. Kinetex Biphenyl (2.1 30 mm, 5 μm particle) column was used where the injection volume was 20 mL. H_2_O containing 0.1% acetic acid or 90:20 MeCN: H_2_O containing 0.1% acetic acid were used differently as mobile phase.

### Statistical analysis

All statistical analysis was performed by one-way ANOVA followed by Dunnett’s test or Turkey test for comparing variable groups using GraphPad prism 5 software. In certain case Student’s *t*-test was also performed. Values are expressed as mean ± SEM for control and treated samples. The values were considered statistically significant as per *p* values (*p < 0.05, **p < 0.01, ***p < 0.001, ns = not significant).

## Electronic supplementary material

supplementary information

Supplementary Figure 1

Supplementary Figure 2

Supplementary Figure 3

Supplementary Figure 4

Supplementary Figure 5

Supplementary Figure 6
