# Supplementary material for: Inhibition of cancer progression by a novel trans-stilbene derivative through disruption of microtubule dynamics, driving G2/M arrest, and p53-dependent apoptosis
Source: Cell Death Dis. 2018 Apr 18;9(5):448. doi: 10.1038/s41419-018-0476-2 (PMC5906627; doi:10.1038/s41419-018-0476-2)
Supplement: Supplementary file 1 — supplementary information [file 41419_2018_476_MOESM1_ESM.docx]

**Supplementary information**

**Inhibition of cancer progression by a novel *trans*-stilbene derivative through disruption of microtubule dynamics, driving G2/M arrest and p53-dependent apoptosis**

**Running Title: Anti-cancer activity of a novel trans-stilbene**

Pravat Kumar Parida^1^, Barun Mahata^1^, Abhisek Santra^1^, Sohini Chakraborty^2^, Zhumur Ghosh^2^, Sanghamitra Raha^3^, Anup Kumar Misra^1^, Kaushik Biswas^1^* and Kuladip Jana^1^*

^1^From the Division of Molecular Medicine, Bose Institute, P1/12, C.I.T. Scheme VIIM, Kolkata, West Bengal, 700054, India, ^2^The Bioinformatics Center, Bose Institute, Kolkata, West Bengal, 700054, India and ^3^Saha Institute of Nuclear Physics, Kolkata, India

* To whom all correspondence should be addressed

Kuladip Jana, Division of Molecular Medicine, Bose Institute, Kolkata, India, Email: [kuladip_jana@yahoo.com](mailto:kuladip_jana@yahoo.com), [kuladip.jana@gmail.com](mailto:kuladip.jana@gmail.com) . Ph. No. 091-9007042850;

Kaushik Biswas, Division of Molecular Medicine, Bose Institute, Kolkata, India,

Email : [kbiswas_1@yahoo.com](mailto:kbiswas_1@yahoo.com). Ph. No. 091-9007191031

**Supplementary Figure legends**

**Figure S1: Typical experimental condition for the preparation of (Z)-2, 3-diarylacrylonitrile (trans-stilbene) derivatives*.*** Briefly, to an ice-cold solution of aryl acetonitriles (1-4) (1 mmol) and aldehydes (5-19) (1.1 mmol) in EtOH (10 mL) was added 5% aq. NaOH (0.2 mL) and the reaction mixture was briskly stirred at room temperature for 30 min. Acidification of the reaction mixture using 1N HCl led to the formation of products as solids. Filtration of the solid products and re-crystallization from EtOH furnished pure products ZDAN-1−ZDAN-22.

**Figure S2: Z-DAN-11 induced G2/M phase cell cycle arrest.** Synchronized (double thymidine blocked) HeLa and MDA-MB-231 cells were treated with with Z-DAN-11 in dose dependent (0, 0.5, 1, 2.5, 5 and 10µM) manner for 24 hrs. Post treatment cells were harvested and fixed with ethanol followed by PI staining to check cycle progression by flow cytometry. Histograms representing dose-dependent increase in G2/M phase accompanied by decrease in G0/G1 phase in **(a)** HeLa and **(b)** MDA-MB-231 cells in response to treatment of Z-DAN-11 **(c)** Treatment of Z-DAN-11 induced mitotic DNA condensation as indicated by increase in phosphorylated form of H3 ser10 in MDA-MB-231 cell line. **(d)** Shows the effect of Z-DAN-11 on α-tubulin, a microtubule protein in MCF-7 and A549 cells. **(e)** Effect of Z-DAN-11 on α- and β- tubulin expression in MDA-MB-231 cells. Images were acquired in Leica confocal microscope (magnification: 63×) One-way ANOVA followed by Dunnett’s t-test was performed to comparisons of multiple group means (treatments) versus DMSO control. Data are representative of atleast three independent experiments and bar graph shows mean ± SEM (*p <0.05**p < 0.01, ***p < 0.001, ns= not significant).

**Figure S3: Validation of differential regulation of genes in response to Z-DAN-11 by q-PCR (a)** Quantitative real time-PCR (q-PCR) validation of some genes that were significantly regulated in microarray in response to Z-DAN-11 in MCF-7 cells. **(b)** Shows comparative fold change of some genes in microarray and q-PCR after 12hrs of Z-DAN-11(10µM) treatment. **(b)** Sequence of primers that are used for q-PCR.

**Figure S4: Depicts effect of Z-DAN-11 on apoptosis. (a)** Apoptosis inducing ability of Z-DAN-11 by flow cytometric analysis of annexin V- FITC/PI staining along with quantitative bar graph in MDA-MB-231 cells. **(b)** Identification of apoptotic nuclei by TUNEL staining in MDA-MB-231 cells. **(c)** DAPI staining showing induction of DNA damage by Z-DAN-11 in MCF-7, A549 and MDA-MB-231 cells. **(d)** Proteome Profiler^™^ Human Apoptosis Array Kit results showing time-dependent differential regulation of 35 apoptosis related protein in response to Z-DAN-11 in MCF-7 cells.

**Figure S5: Z-DAN-11 induced ROS production contributes to mitochondrial dysfunction and cancer cell apoptosis.**

**(a)** Microscopic images of JC1 staining in A549 cells indicating Z-DAN-11 induced time- and concentration-dependent increase in the mitochondrial permeability which is evident from the red to green shift of fluorescence. **(b)** Flow cytometric detection of cellular ROS by DCFDA method. Briefly, cells were treated with test compounds (0, 2.5,5 and 10 μM) for 6 and 9 hours. and then DCFDA (5µM final) was added and incubated for 30 mins. Post incubation the media was discarded and adherent cells were scrapped out and washed in 1×PBS. Finally the fluorescent signals from the cells were acquired by FACS-Verse. **(c)** MitoSOX™ Red staining microscopic data revealed Z-DAN-11 induces mitochondrial ROS production. Post treatment cells with coverslips were fixed with 3.7% formaldehyde, washed with 1×PBS and DAPI staining was done and washed with 1×PBS. Coverslips with stained cells was mounted on slides and observed in Leica confocal microscope. **(d)** Confocal microscopic images showing protection of Z-DAN-11 mediated mitochondrial ROS by N-acetyl-cysteine (NAC). Briefly, 5×10^4^A549 cells grown on cover slips were pre-treated with 10mM ROS scavenger NAC for 4hrs followed by treatment with 10µM of Z-DAN-11 for 6hrs and 9hrs were fixed with 3.7% formaldehyde and washed with 1×PBS. DAPI staining was done and washed with 1× PBS. Coverslips with stained cells were mounted on slides and observed in Leica confocal microscope. (**e)** Demonstrates the involvement of ROS in Z-DAN-11 mediated mitochondrial dysfunction in A549 cells apoptosis. Briefly, A549 cells were pre-treated with 10mM ROS scavenger NAC for 4hrs followed by treatment with 10µM of Z-DAN-11 for 48hrs. Finally cells were trypsinized, stained with Annexin V-FITC/PI and analyzed by flow cytometry. Bar graph shows mean ± SEM (*p <0.05**p < 0.01, ***p < 0.001, ns= not significant).

**Figure S6: Showing hepatotoxic effect and absolute bioavailability of Z-DAN-11 (a)** Effect of Z-DAN-11 on body weight of mice and serum ALT, AST, ALP, creatinine, urea and nitrogen. Following seven doses Z-DAN-11(10mg/kg and 20mg/kg) each with three days interval, 100μl blood sample were taken manually into heparinized capillary tubes by piercing the saphaneous vein with a needle and subsequently into 0.5 ml microcentrifuge tubes. All blood samples were processed for plasma isolation by centrifugation at 1640 rpm for 5 min at 4^o^C within half an hour of collection. All the above data are representative of two independent experiments and bar graph shows mean ± SEM (*p <0.05**p < 0.01, ***p < 0.001, ns= not significant). **(b)** Represents time-dependent change in mean plasma concentration of Z-DAN-11 following intravenous and oral administration. Briefly, isolated plasma samples at each time point, post oral and intravenous dose of Z-DAN-11 was analyzed using Sciex 5500 Q-Trap LC-MS/MS and agilent 1290 series high performance liquid chromatograph. Kinetex Biphenyl (2.1 30 mm, 5 μm particle) column was used where the injection volume was 20 ml. H2O containing 0.1% Acetic Acid or 90:20 MeCN: H_2_O containing 0.1% Acetic Acid were used differently as mobile phase. Data were shown as (mean± S.D., n=3).

**Table S1:** Showing the comparative IC_50_ doses of the synthesized derivatives and Resveratrol in HeLa, MCF-7 and NKE cells.

| Compounds | HeLa IC_50_(µM) | NKE CC_50_ (µM) | MCF-7 IC_50 (_µM) | SI(NKE/MCF-7) | SI(NKE/HeLa) |
| --- | --- | --- | --- | --- | --- |
| Z-DAN-1 | **12.79±7.24** | --- | 14.36 ± 8.89 | --- | --- |
| Z-DAN-2 | 24.61±6.27 | 34.1 ± 10.07 | 19.56 ± 9.22 | 1.93±0.39 | 1.37±0.06 |
| Z-DAN-3 | 20.46±4.21 | 26.58±9.41 | 26.31±4.69 | 0.98±0.18 | 1.26±0.20 |
| Z-DAN-4 | 37.18±14.86 | 11.24±6.44 | 27.26±5.79 | 0.38±0.16 | 0.28±0.06 |
| Z-DAN-5 | **12.68±6.51** | **38.62 ± 27.59** | >60 | <1 | **2.62±0.83** |
| Z-DAN- 6 | 28.93 ± 3.14 | 38.37 ± 8.18 | 37.59 ± 9.21 | 1.03±0.03 | 1.31±0.14 |
| Z-DAN-7 | 46.64±7.58 | 29.42±5.63 | 16.74±5.31 | 1.84±0.25 | 0.63±0.02 |
| Z-DAN-8 | >60 | 31.29±5.62 | 18.50±2.53 | 1.68±0.07 | <1 |
| Z-DAN-9 | --- | --- | 18.83±5.86 | --- | --- |
| Z-DAN-10 | **6.29±1.8** | 28.02±5.74 | 27.80±8.16 | 1.04±0.10 | **4.57±0.39** |
| Z-DAN-11 | **4.96±0.32** | **40.24 ± 5.48** | **5.52±0.43** | **7.26±0.43** | **8.08±0.58** |
| Z-DAN-12 | **8.20 ±2.33** | **33.71 ± 8.5** | **8.29 ± 1.97** | **4.05±0.06** | **4.15±0.14** |
| Z-DAN-13 | --- | --- | 42.31±8.07 | --- | --- |
| Z-DAN-14 | >60 | 43.15 ± 8.64 | 23.2 ± 10.27 | **2.11±0.56** | <1 |
| Z-DAN-15 | 22.47±8.62 | 29.04±7.75 | 49.89±4.69 | 0.57±0.10 | 1.36±0.18 |
| Z-DAN-16 | 17.58±9.58 | 24.75 ±8.41 | 34.99±14.92 | 0.74±0.07 | 1.63±0.41 |
| Z-DAN-17 | 14.87±6.57 | 35.88 ±16.52 | 21.9 ± 9.56 | 1.62±0.05 | **2.39±0.06** |
| Z-DAN-18 | 31.17±9.07 | 28.57±5.96 | 32.36±8.20 | 0.89±0.04 | 0.94±0.08 |
| Z-DAN-19 | 19.45±7.35 | 34.61 ± 8.87 | 13.25 ± 4.6 | **2.71±0.27** | 1.87±0.25 |
| Z-DAN-20 | >60 | 54.36±14.27 | --- | --- | <1 |
| Z-DAN-21 | 25.73±12.95 | 33.15±2.81 | 9.27±2.14 | **3.7±0.55** | 1.65±0.72 |
| Z-DAN-22 | --- | 9.24±3.78 | 10.92±3.19 | 0.82±0.11 | --- |
| Resveratrol | 36.81±3.26 | 43.98±8.37 | 40.6±15.96 | 1.19±0.26 | 1.18±0.12 |

IC_50_=50% inhibitory concentration in cancer cells, CC_50_=50% cytotoxic concentration in normal cells, SI=Selectivity index (CC_50_/ IC_50_)
