# Supplementary figures and images for: Inhibition of cancer progression by a novel trans-stilbene derivative through disruption of microtubule dynamics, driving G2/M arrest, and p53-dependent apoptosis
Source: Cell Death Dis. 2018 Apr 18;9(5):448. doi: 10.1038/s41419-018-0476-2 (PMC5906627; doi:10.1038/s41419-018-0476-2)

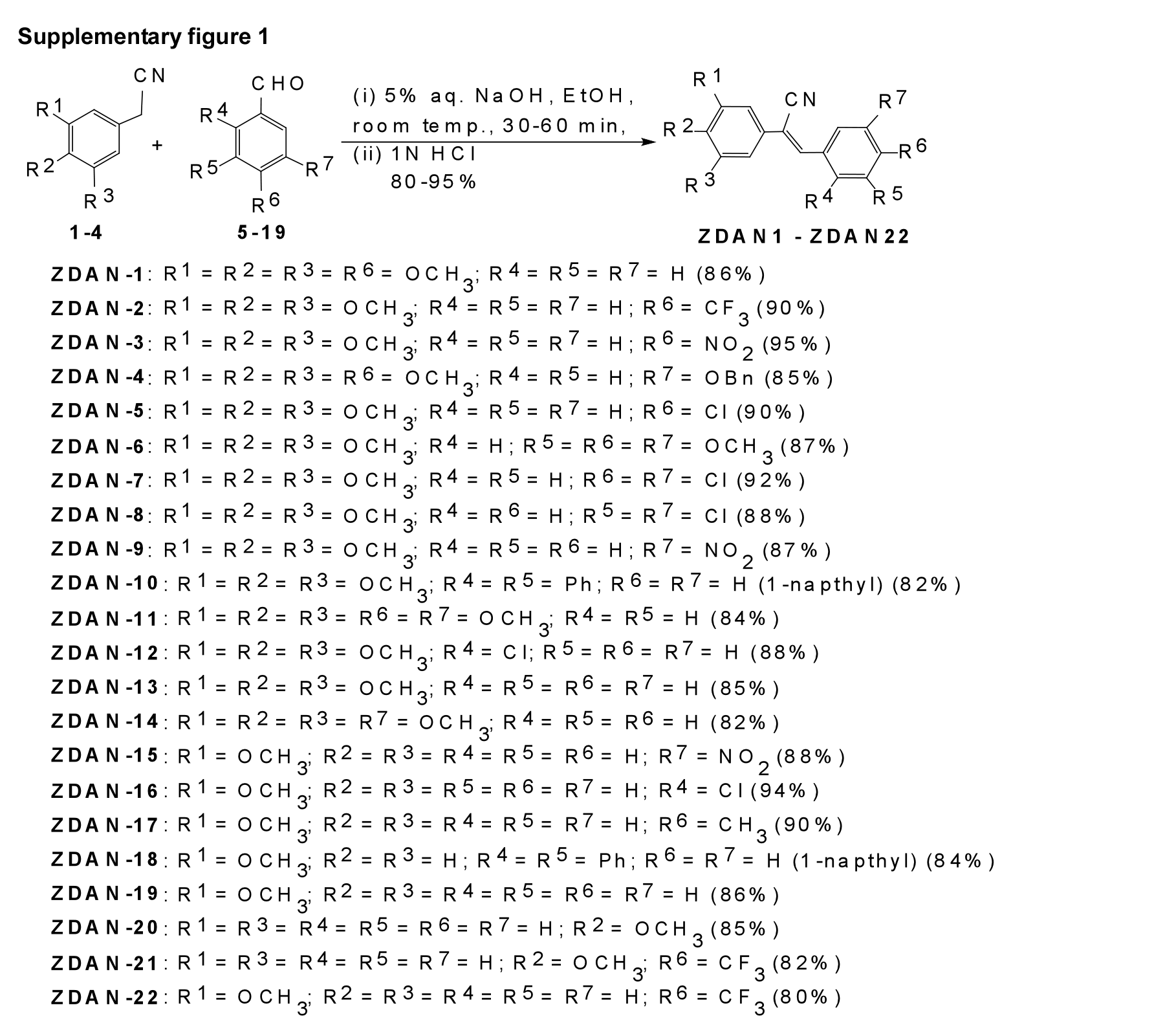

Supplement: Supplementary file 2 — Supplementary Figure 1 [file 41419_2018_476_MOESM2_ESM.tif]

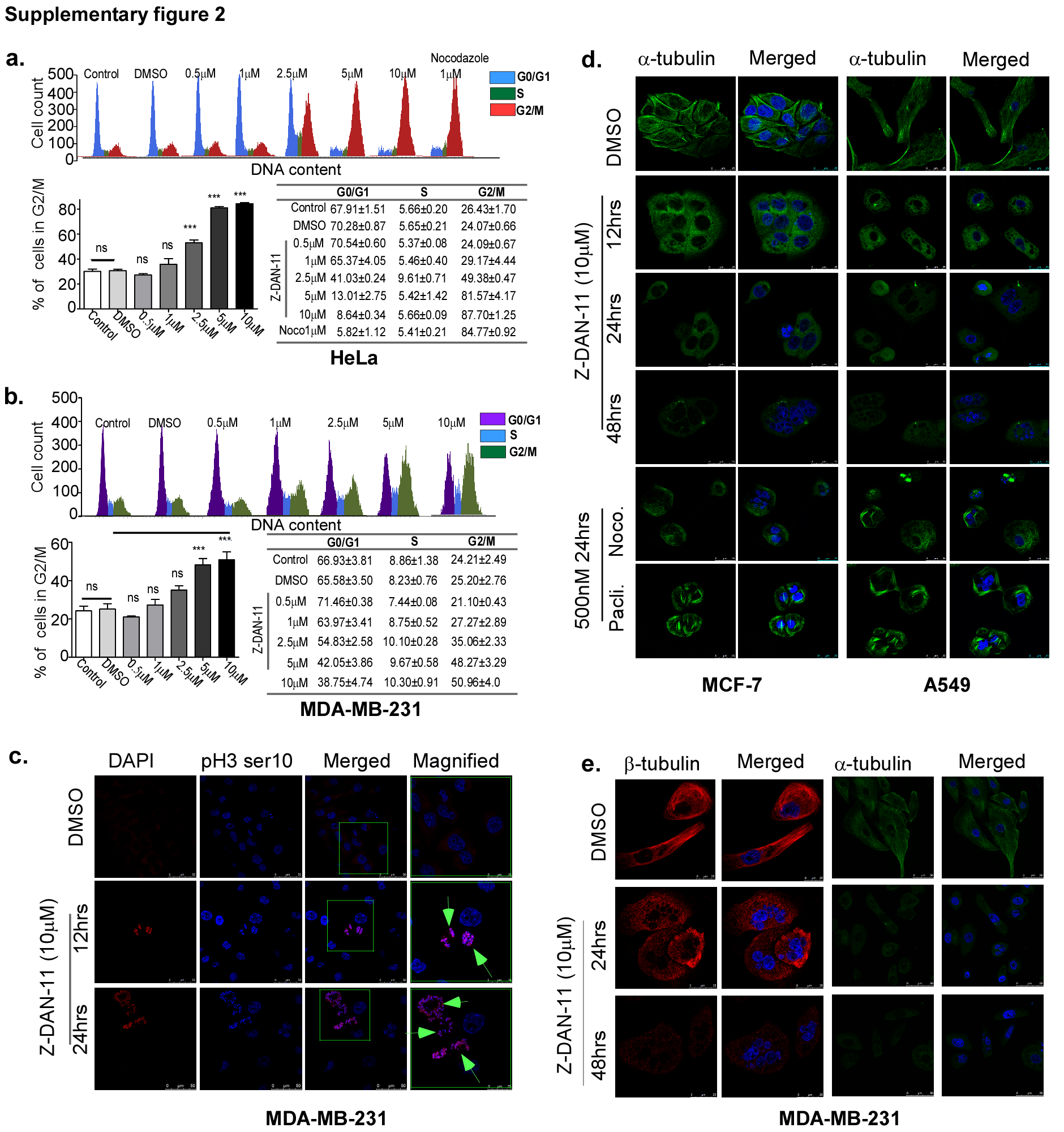

Supplement: Supplementary file 3 — Supplementary Figure 2 [file 41419_2018_476_MOESM3_ESM.tif]

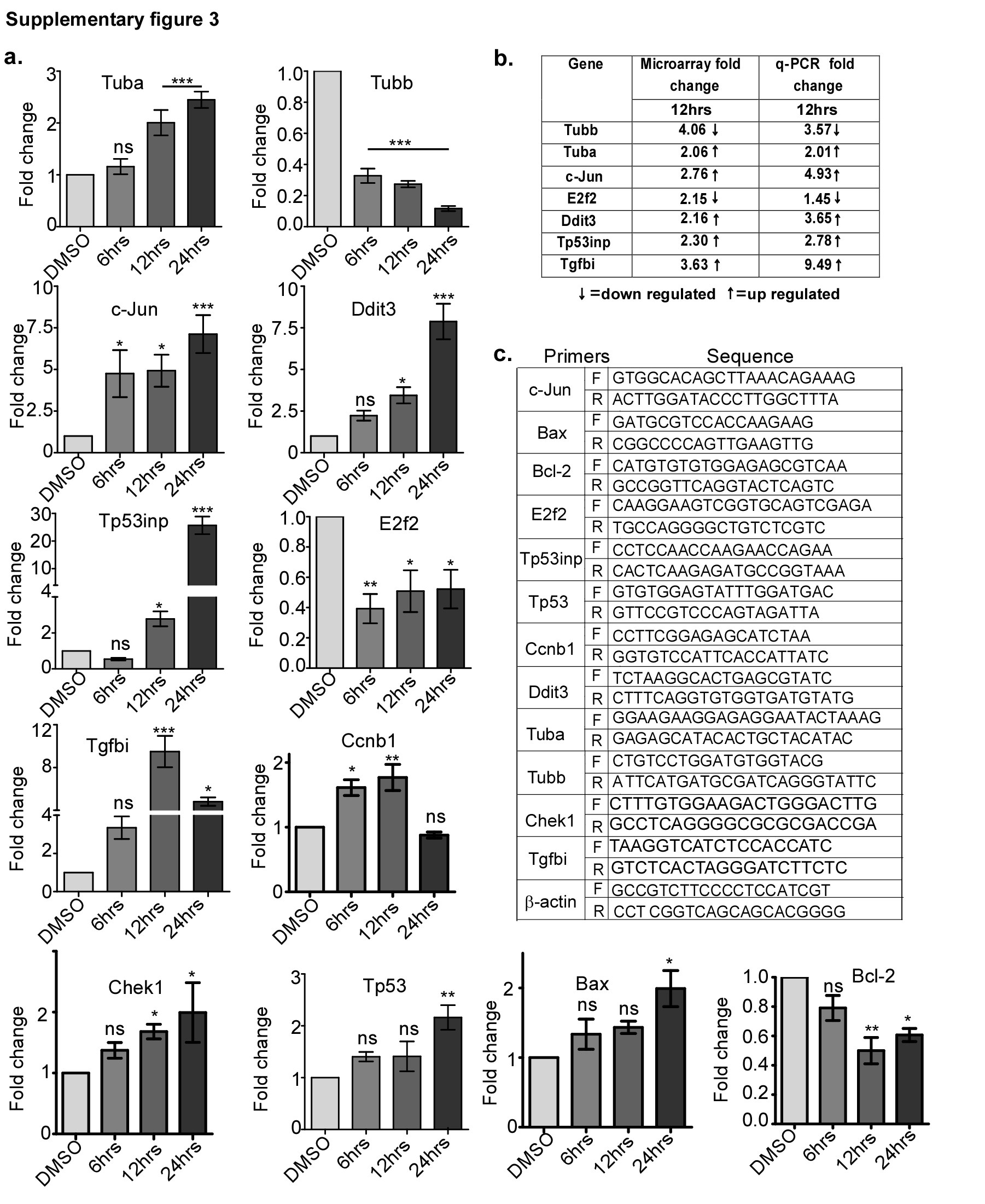

Supplement: Supplementary file 4 — Supplementary Figure 3 [file 41419_2018_476_MOESM4_ESM.tif]

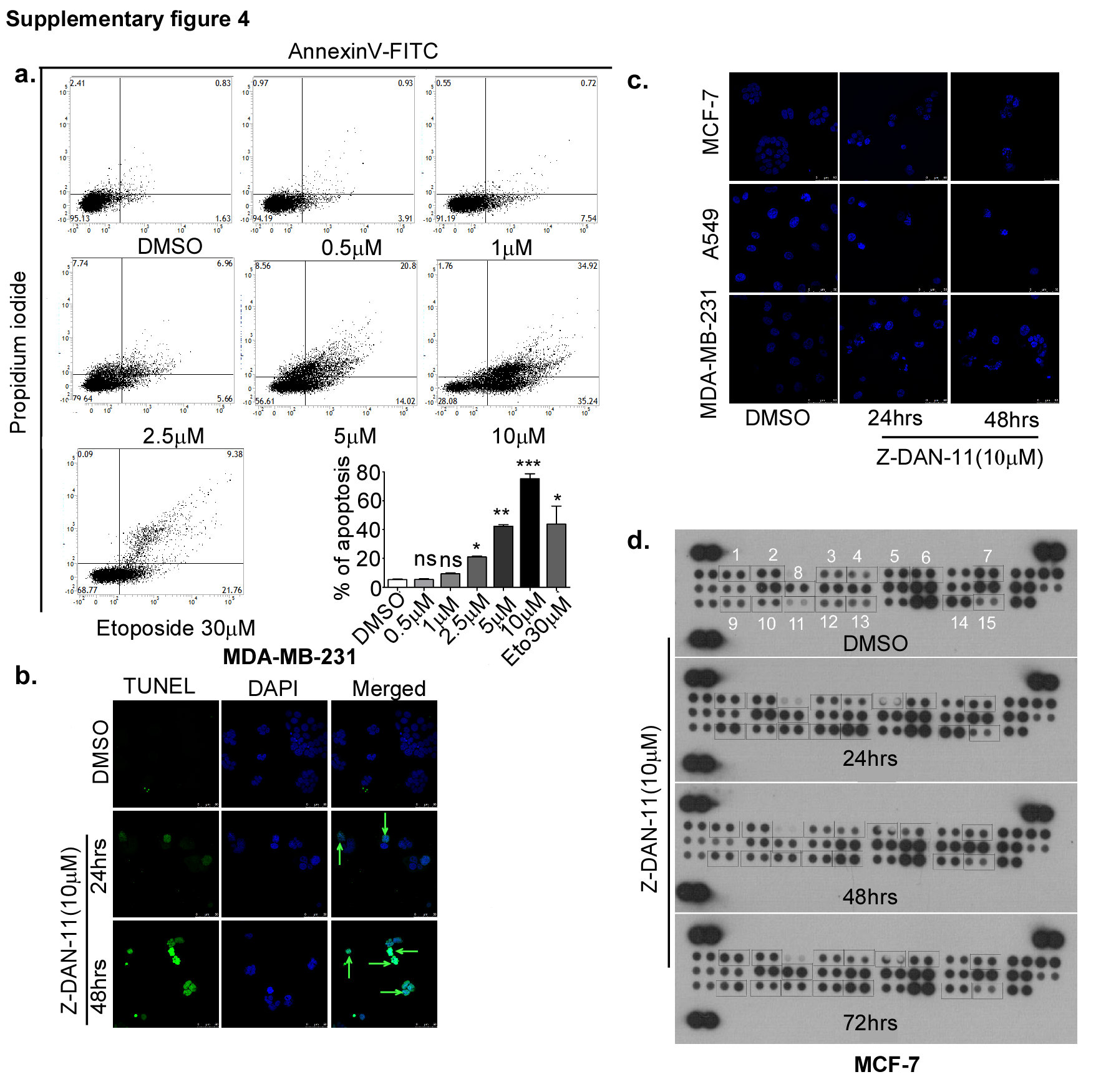

Supplement: Supplementary file 5 — Supplementary Figure 4 [file 41419_2018_476_MOESM5_ESM.tif]

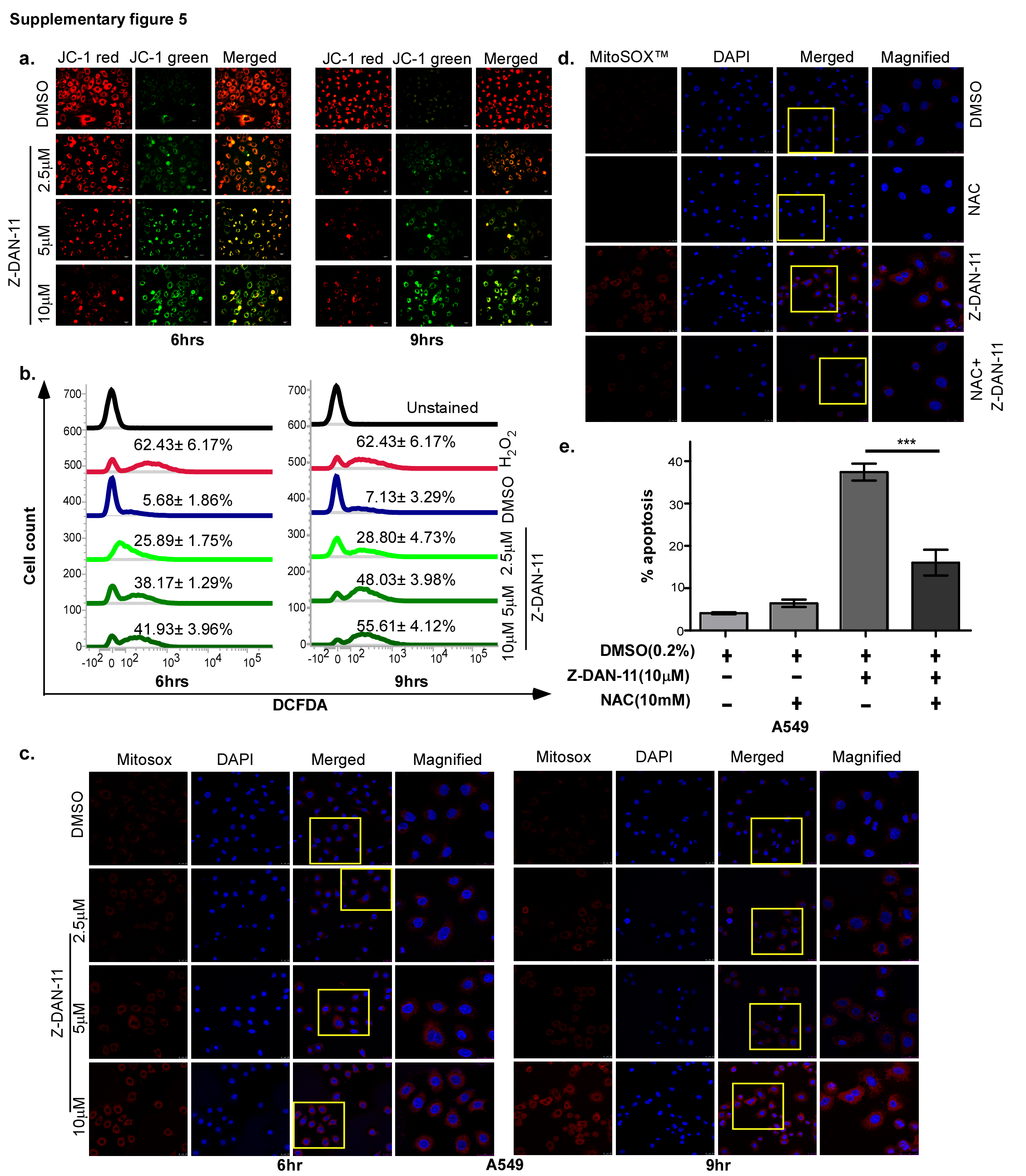

Supplement: Supplementary file 6 — Supplementary Figure 5 [file 41419_2018_476_MOESM6_ESM.tif]

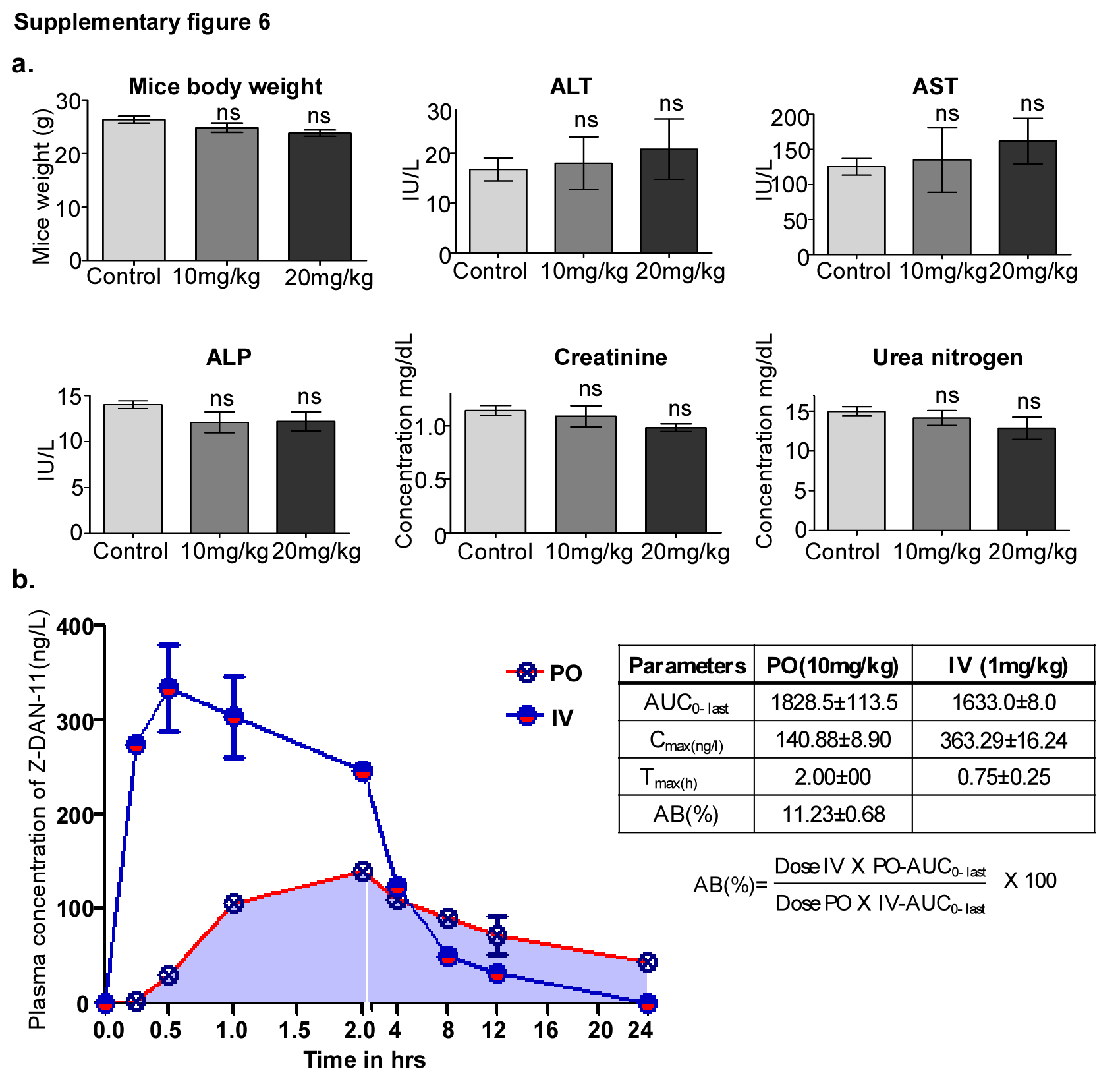

Supplement: Supplementary file 7 — Supplementary Figure 6 [file 41419_2018_476_MOESM7_ESM.tif]
